# miR146a-mediated targeting of FANCM during inflammation compromises genome integrity

**DOI:** 10.18632/oncotarget.10275

**Published:** 2016-06-24

**Authors:** Devakumar Sundaravinayagam, Hye Rim Kim, TingTing Wu, Hyun Hee Kim, Hyun-Seo Lee, Semo Jun, Jeong-Heon Cha, Younghoon Kee, Ho Jin You, Jung-Hee Lee

**Affiliations:** ^1^ Laboratory of Genomic Instability and Cancer Therapeutics, Cancer Mutation Research Center, Chosun University School of Medicine, Gwangju, Republic of Korea; ^2^ Department of Pharmacology, Chosun University School of Medicine, Gwangju, Republic of Korea; ^3^ Department of Cellular and Molecular Medicine, Chosun University School of Medicine, Gwangju, Republic of Korea; ^4^ Department of Oral Biology, Department of Applied Life Science, The Graduate School, Yonsei University College of Dentistry, Seoul, Republic of Korea; ^5^ Department of Cell Biology, Microbiology, and Molecular Biology, College of Arts and Sciences, University of South Florida, Tampa, Florida, United States of America

**Keywords:** miR146a, FANCM, Fanconi anemia pathway, DNA interstrand cross-links (ICLs) repair, NF-κB

## Abstract

Inflammation is a potent inducer of tumorigenesis. Increased DNA damage or loss of genome integrity is thought to be one of the mechanisms linking inflammation and cancer development. It has been suggested that NF-κB-induced microRNA-146 (miR146a) may be a mediator of the inflammatory response. Based on our initial observation that miR146a overexpression strongly increases DNA damage, we investigated its potential role as a modulator of DNA repair. Here, we demonstrate that FANCM, a component in the Fanconi Anemia pathway, is a novel target of miR146a. miR146a suppressed FANCM expression by directly binding to the 3′ untranslated region of the gene. miR146a-induced downregulation of FANCM was associated with inhibition of FANCD2 monoubiquitination, reduced DNA homologous recombination repair and checkpoint response, failed recovery from replication stress, and increased cellular sensitivity to cisplatin. These phenotypes were recapitulated when miR146a expression was induced by overexpressing the NF-κB subunit p65/RelA or *Helicobacter pylori* infection in a human gastric cell line; the phenotypes were effectively reversed with an anti-miR146a antagomir. These results suggest that undesired inflammation events caused by a pathogen or over-induction of miR146a can impair genome integrity via suppression of FANCM.

## INTRODUCTION

Chronic inflammation is believed to be a causative factor for tumorigenesis. NF-κB is a crucial regulator of the inflammatory response. Its constitutive or transient activation enables genetic alterations and oncogene activation, and confers cancer-promoting potential to cells [1]. NF-κB forms multiple homodimeric- or heterodimeric complexes with Rel-like domain-containing proteins, including RelA/p65. It is a pleiotropic transcription factor that can exert profound effects on the cellular immune state by coordinating the expression of numerous downstream targets [2, 3]. MicroRNA-146a (miR146a), recognized as a gene targeted by NF-κB, has been implicated in a variety of cellular functions, particularly in pathological conditions, such as inflammation and cancer [4–6]. Chronic inflammation caused by a variety of pathogens such as *Helicobacter pylori (H. pylori)* can presumably induce the long term expression of NF-κB and miR146a [7–9]. This study investigated the potential influence of *H. pylori* infection, activation of NF-κB, and miR146a on cellular genome integrity and tumorigenesis.

MicroRNAs (miRNAs) are small non-coding RNAs with approximately 20–24 nucleotides in length that repress gene expression, usually by targeting the 3′-untranslated regions (3′UTRs) of specific mRNAs. Over the past decade, *in silico* screening of the sequencing database combined with reporter gene-based assays has led to the discovery of miRNAs targeting genes of interest. Mounting evidence suggests that the functions of a number of key factors involved in DNA repair are also regulated by miRNAs.

The Fanconi anemia (FA)-associated DNA damage response pathway is a crucial DNA repair mechanism that resolves DNA interstrand crosslinked (ICL) lesions and stalled replication forks [10]. A key regulatory event in the pathway is the monoubiquitination of Fanconi anemia complementation group D2 (FANCD2), which is induced by an E3 ubiquitin ligase complex consisting of 8 FA proteins, including Fanconi anemia group M protein (FANCM) [11]. Monoubiquitinated FANCD2 forms foci at damaged lesions that co-localizes with DNA repair factors such as FANCI, BRCA1, and RAD51 [12]. Among the several reported roles of monoubiquitinated FANCD2 is recruiting the carboxy-terminal binding protein-interacting protein (CtIP) nuclease, which induces end resection at double strand breaks, a step required for DNA homologous recombination repair (HR) and replication fork recovery [13–15]. FANCD2 is also essential in the removal and bypass of ICL-lesions by DNA replication machineries [16]. In addition to being part of the FA core complex, FANCM forms a complex with FA-associated protein 24 KDa (FAAP24)-MHF1-MHF2, each of which is required for recruitment of the FA core complex at chromatin and FANCD2 monoubiquitination. FAAP24 is required for the DNA-binding and chromatin loading abilities of FANCM *in vitro* [17]. FANCM also exhibits ATP-dependent replication fork remodeling and branch migration activities using model fork structures *in vitro*, which may be needed for stabilizing DNA replication forks during the ICL repair [18, 19]. The fork remodeling and branch migration activities are enhanced by MHF1-MHF2 *in vitro* [20–22]. The FANCM-FAPP24 complex also plays an important role in regulating ICL-induced, ATR-mediated checkpoint [23, 24]. The roles of FANCM likely extend beyond the canonical FA pathway, suggested by the roles of FANCM orthologs in other organisms; additional information on this topic is summarized in a recent review article [25]. Overall, FANCM plays multiple roles in genome maintenance during replication and DNA repair.

Here, we show that FANCM stability is directly regulated by miR146a. The overexpression of miR146a or artificial activation of NF-κB drastically suppressed FANCM expression, and was associated with impaired damage-inducible FANCD2 monoubiquitination, HR repair, and increased cellular sensitivity to hydroxyurea (HU) and cisplatin. The physiological significance of this pathway was supported by the observation that miR146a expression was induced by *H. pylori* infection, which led to reduced FANCM expression and FANCD2 foci formation. These data suggests the aberrant activation of inflammation-induced miR146a can compromise genome integrity by suppressing FANCM expression.

## RESULTS

### miR146a directly targets the 3′UTR of FANCM

**Figure 1:**
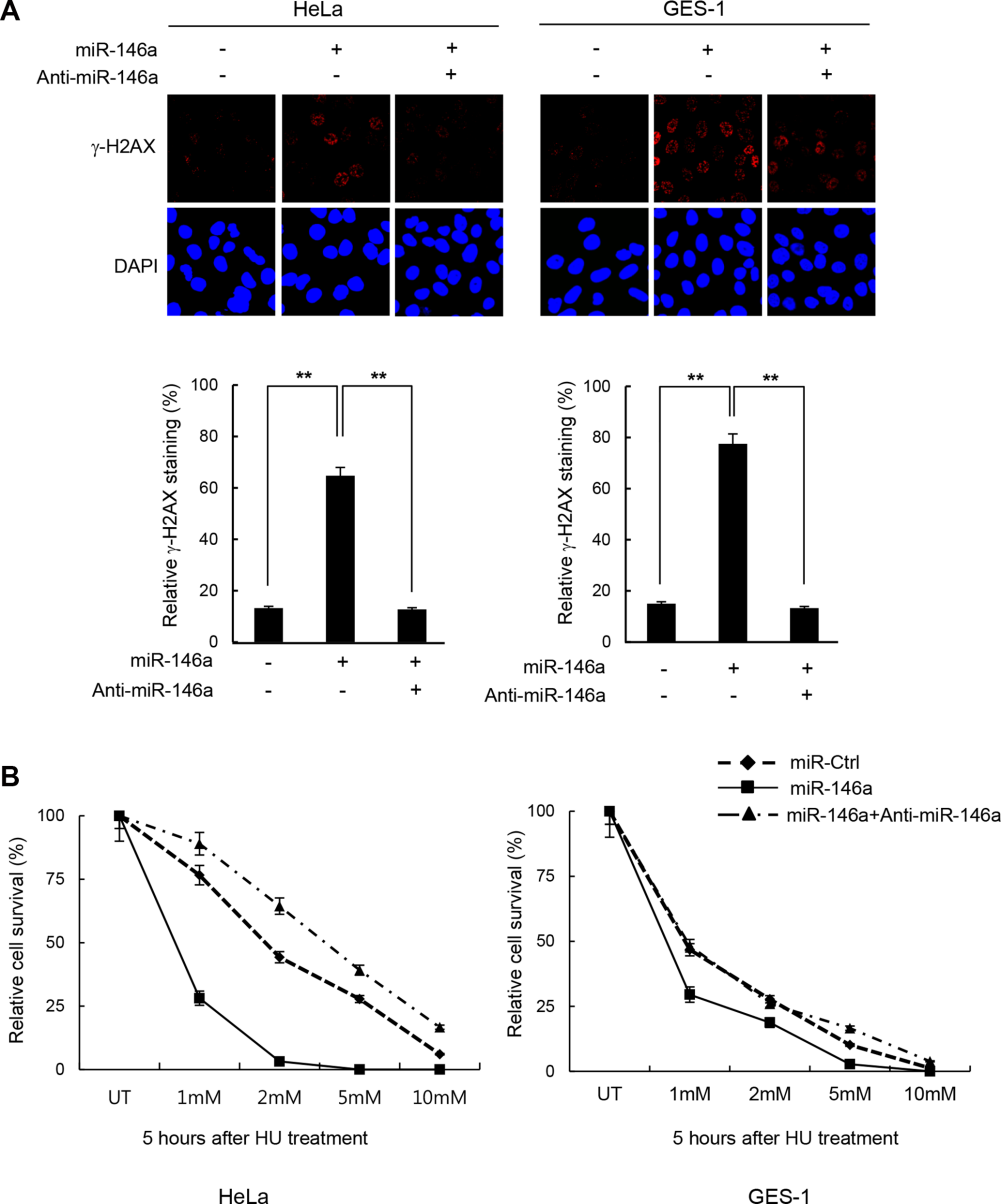
Increase of DNA damage sensitivity by miR146a (**A**) Two days after transfection of HeLa or GES-1 cells with miR146a alone or together with anti-miR146a, the cells were treated with 5 mM HU for 16 h to detect residual γ-H2AX. Results are shown as the mean ± SD (*n* = 3); **P < 0.01. (**B**) HeLa and GES-1 cells were transfected with control, miR146a, and miR146a plus anti-miR146a, and were then exposed to increasing concentrations of HU for 5 hr. The viability of treated cells was examined using the clonogenic survival assay. Results are shown as the mean ± SD (*n* = 3); **P < 0.01.

It is becoming well-accepted that inflammation-induced miR146a is associated with cancer development [4–6, 26]. Our initial analysis of miR146a showed that its overexpression strongly elicited DNA damage in HeLa (human cervical adenocarcinoma) and GES-1 cells (human gastric epithelial cells) upon treatment with the replication stress inducer HU and the DNA ICL-inducing agent cisplatin, as measured by γ-H2AX foci staining (Figure 1A and Supplementary Figure S1A, respectively). Consistent with the increased damage, cells expressing miR146a were more sensitive to HU and cisplatin (Figure 1B and Supplementary Figure S1B, respectively). Moreover, co-expression of miR-146a antagomir effectively reversed these phenotypes, suggesting that these effects were indeed mediated by miR146a. Together, these data suggest that overexpressing miR146a may impair genome integrity.

In an attempt to search for the relevant targets of miR146a in DNA Damage Response (DDR) or DNA repair, we utilized the TargetScan algorithm (MIT; release 6.2). This method identified several DDR genes as potential targets of miR146a, including BRCA1 and FANCM. Indeed, BRCA1 was previously identified as a miR146a target [27], validating our bioinformatic approach. To determine whether their effect of miR-146a on DNA damage response was mediated via its effect on FANCM, we cotransfected HeLa and GES-1 cells with miR-146a and/or miR-146a-insensitive FANCAM expression plasmid. Our results showed that the non-targetable FANCM rescued the HU-induced γ-H2AX foci formation (Supplementary Figure S2A) and cellular sensitivity to HU (Supplementary Figure S2B). We also observed similar reversion when miR-146a-insensitive BRCA1 was expressed. Interestingly, when we expressed both FANCM and BRCA1 simultaneously, we did not see further rescue, indicating that FANCM and BRCA1 may have overlapping roles in the phenotype that we are seeing.

**Figure 2:**
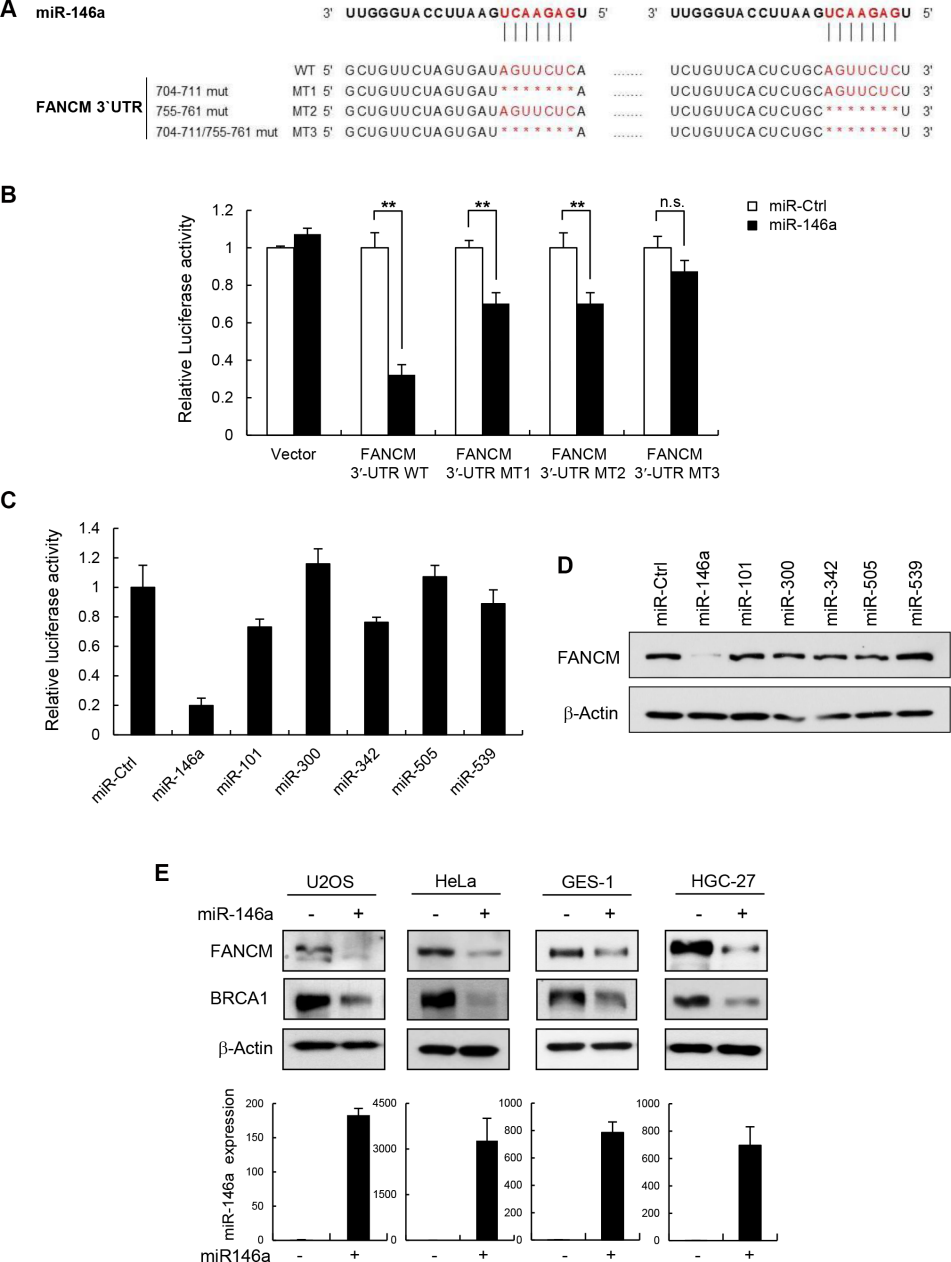

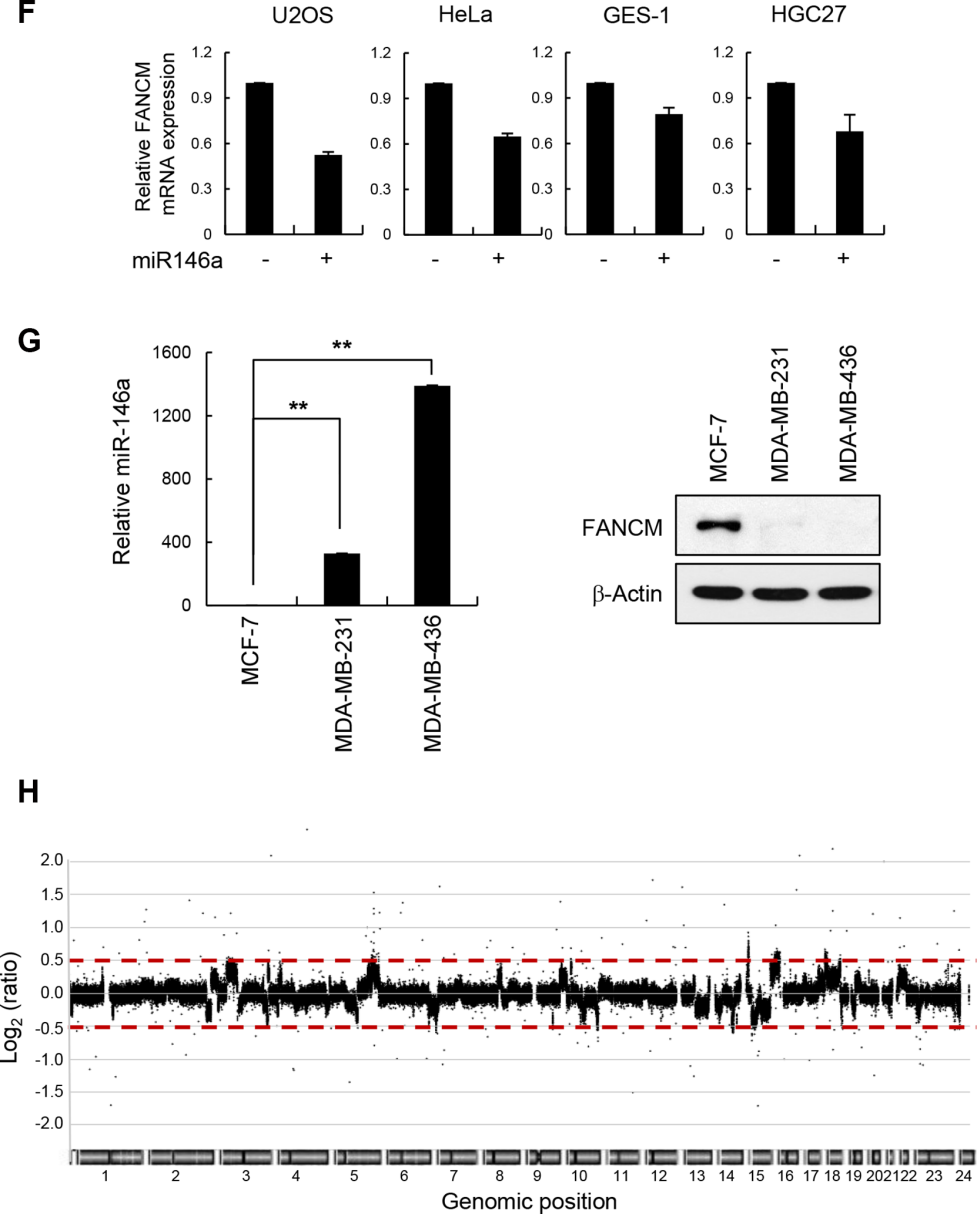
miR146a directly targets FANCM (**A**) Schematic diagrams show the sequence and position of predicted miR146a binding sites in the FANCM 3′UTR. Single- (M1 and M2) and double- (M3) deletion mutations of the miR146a target sites were shown. The seed resion of miR146a is in red. (**B**) FANCM 3′UTR-WT and FANCM 3′UTR-MT1-3 were cotransfected with miR146a into HeLa cells. Luciferase activity was measured 24 h after transfection. Data represent the mean ± SD (n = 3); **P < 0.01. (C) HeLa cells were cotransfected with the FANCM 3′UTR luciferase reporter vector along with the candidate miRNAs, which were predicted by Targetscan v6.2, or the miRNA-negative control (miR-Ctrl). Results are shown as the mean ± SD (*n* = 3). (**D**) The levels of FANCM protein were measured by western blotting after transfection of miR146a into a number of cell lines as indicated. (**E**) Western blot analysis using cell extracts was performed to detect cellular levels of FANCM and BRCA1 after transfecting indicated cells with miR146a (*top*). In parallel, the transfected cells were subjected to RNA extraction, followed by RT-qPCR to measure the amount of miR146a after transfection. The relative amount of miR146a was quantified and shown as the mean ± SD (n = 3); **P < 0.01 (*bottom*). (**F**) Expression of *FANCM* mRNA in miR146a transfected cells was quantitated using real-time qPCR. Results are shown as the mean ± SD (*n* = 3); **P < 0.01. (**G**) Three different breast cancer cell lines, MCF7, MDA-MB-231, and MDA-MB-436, were cultured and cell extracts were analyzed for evaluating miR146a (*left*) and FANCM expression (*right*) using Western blotting and RT-qPCR, respectively. Results are shown as the mean ± SD (*n* = 3); **P < 0.01. (**H**) Array CGH profiles of human fibroblast GM00637 cells transfected with miR146a versus control miRNA. Chromosomal regions above or below the red dotted line indicate gains or losses of corresponding genomic positions, respectively.

Subsequent TargetScan analysis predicted that miR146a targeted two sequences found between nucleotides (nt) 704 and 711 and between nt 755 and 761 on the human FANCM 3′UTR sequence (Figure 2A). To confirm the interaction of miR146a with these predicted sites and the subsequent repression of FANCM, we performed a 3′UTR luciferase reporter assay. The relative luciferase activity of the construct with wild-type 3′UTR of FANCM significantly repressed following the transfection of miR146a (Figure 2B). In contrast, the repression of relative luciferase activity was clearly abrogated when single (M1 and M2) and especially double (M3) conserved sites were deleted in the FANCM 3′UTR. This indicates that these two seed regions are functional as miR146a targets sites, exerting additive effects on each other. Accordingly, the MT3 mutant was shown to be nonfunctional as a target of miR146a due to the complete loss of both target sites.

Next, to examine the evolutionary conservation of the identified target sites across species, the human FANCM 3′UTR of 878 nt in length (GenBank accession number: NM_020937.3) was aligned with 100 vertebrate species using the algorithms provided by the UCSC genome browser (https://genome.ucsc.edu/index.html). Surprisingly, alignment data showed that the FANCM 3′UTR was only conserved between primates, and that sequence differences with the other species were such that full sequence alignment was not allowed. While the two target sites of miR146a was relatively well conserved between primates (Supplementary Figure S3, red open box), neither target site was detected in species other than primates. These results suggest that the miR146a-mediated regulation of FANCM expression was introduced to the primate-specific FA pathway as a relatively late event in vertebrate evolution.

TargetScan algorithm (MIT; release 6.2) was further used to generate a selective miRNA library for screening (Supplementary Table S1). The analysis identified a total of six miRNAs as candidates, each of which was subjected to reverse screening for gene silencing effects on FANCM expression using a luciferase reporter gene assay (Figure 2C). The results showed that miR146a expression led to remarkably lower luciferase activity compared to control miRNA, whereas weak or non-statistically significant effects were observed with the other tested miRNAs. Consistently, miR146a overexpression significantly reduced endogenous FANCM protein level in HeLa cells (Figure 2D). The effects of miR146a on endogenous FANCM expression was further confirmed by comparing its protein levels in two human cancer cell lines, HeLa and U2OS (osteosarcoma) cells, and two human gastric cell lines, HGC-27 (carcinoma) and GES-1 (immortalized normal) cells, after transfecting the cells with miR146a or a negative control miRNA. As shown in Figure 2E, miR146a expression, which was also confirmed using RT-qPCR (Figure 2E, bottom), led to dramatic decreases in FANCM protein levels in all of the tested cells. BRCA1 protein expression was also downregulated, consistent with data from a previous study [27]. Moreover, expressing anti-miR146a in MCF7, HeLa and GES-1 cells led to increases in both FANCM and BRCA1 levels (Supplementary Figure S4). *FANCM* mRNA expression was also reduced when miR146a was overexpressed (Figure 2F), indicating that miR146a post-transcriptionally downregulates FANCM. These results demonstrate that miR146a down-regulates FANCM expression by directly targeting the 3′UTR of its transcript.

### miR146a expression inversely correlates with FANCM protein levels in several breast cancer cell lines

Garcia et al. [27] reported that miR146a was abnormally overexpressed in several breast cancer cells. We chose three of the cell lines described in that report to compare the expression patterns of endogenous miR146a and FANCM protein using RT-qPCR and Western blot analysis, respectively. While the MCF-7 and MDA-MB-231 cell lines express wild type *BRCA1*, MDA-MB-436 cells carry mutated BRCA1 (5396 + 1G > A in the splice donor site of exon 20) (http://research.nhgri.nih.gov/bic/) [28] Western blot analysis of FANCM expression showed that its protein levels were significantly lower in the MDA-MD-231 and MDA-MB-436 cell lines compared to the MCF-7 control cell line (Figure 2G). These results suggest that miR146a expression in breast cancer cell lines is inversely correlated with FANCM protein levels.

When DNA repair does not properly operate in response to DNA ICLs or replication stress, it frequently gives rise to genome-wide chromosomal breaks, leading to genomic instability. To examine the effect of miR146a on genomic integrity, array comparative genomic hybridization was performed using human fibroblast GM00637 cells after transfecting with miR146a or a control miRNA. We found that chromosomal abnormalities were detected as chromosomal gains (dots over 0.5) and losses (dots below −0.5) that were widely distributed throughout the entire genome of miR146a-expressing cells (Figure 2H and Supplementary Figure S5), indicating that overexpressing miR146a causes chromosomal instability.

### miR146a targeting FANCM impairs FANCD2 monoubiquitination

**Figure 3:**
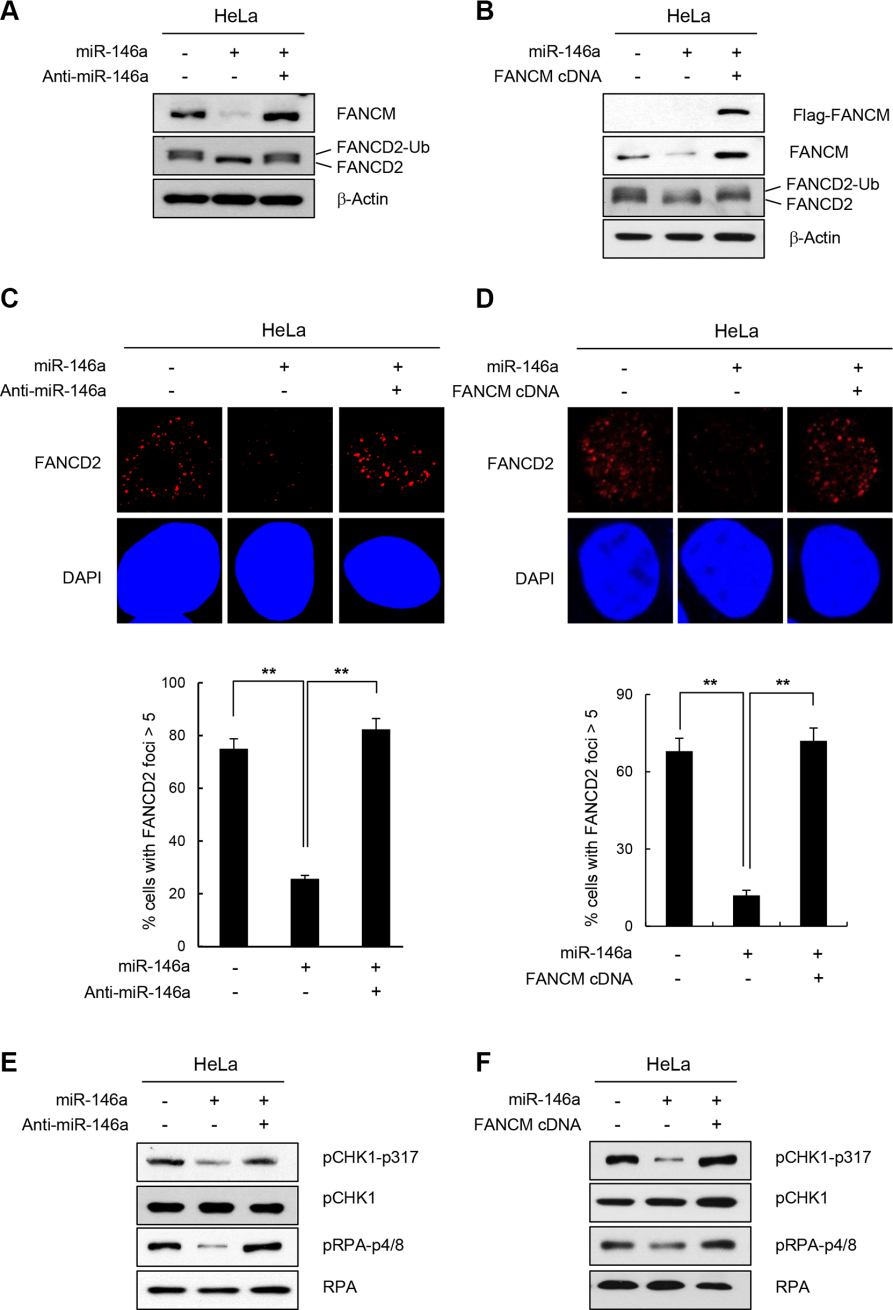
Effect of miR146a on FANCD2 monoubiquitination and the FA pathway (**A** and **B**) HeLa cells were transfected with miR146a in the absence or presence of anti-miR146a (A) or miR146a-insensitive FANCM cDNA (B). After a 48 h transfection, the cells were treated with 5 mM HU for 5 h. The protein levels of FANCM and FANCD2 were measured by western blotting. Monoubiquitinated FANCD2 is indicated as the upper band of doublet protein bands corresponding to FANCD2. (**C** and **D**) After HeLa cells underwent the same treatment as (A and B) cells were analyzed for FANCD2 foci formation. DAPI was used for nuclear staining. Results are shown as the mean ± SD (*n* = 3); **P < 0.01. (**E** and **F**) Indicated cells were treated with 5 mM HU for 5 hr. Cell lysates were analyzed by Western blotting with antibodies against pCHK1-S317, CHK1, pRPA-S4/8, and RPA.

Loss of FANCM leads to reduced FANCD2-ubiquitination [17, 29]. Therefore, we assessed whether overexpressing miR146a interferes with the monoubiquitination of FANCD2 in HeLa and GES-1 cells following treatment with HU, which causes replication stress and activates the FA pathway. Consistent with the reduction in FANCM levels, miR146a expression in HeLa or GES-1 cells led to a significant decrease in monoubiquitinated FANCD2 (FANCD2-Ub) level, compared to a control miRNA (Figure 3A and Supplementary Figure S6A, respectively). The effects of miR146a were effectively reversed by co-transfecting anti-miR146a (Figure 3A), or by expressing exogenous FANCM cDNA that does not harbor the miR146a-binding 3′UTR (Figure 3B), suggesting that the effects are specific to miR146a and FANCM. Consistent with these results, both HeLa and GES-1 cells transfected with miR146a exhibited defective formation of FANCD2 foci compared to cells containing control miRNA, as shown by the decreased number of cells with foci (>5 foci) and these effects were rescued by anti-miR146a or FANCM cDNA expression (Figure 3C, 3D and Supplementary Figure S6B). These findings suggest that reduced FANCM caused by miR146a leads to impaired FANCD2 monoubiquitination and FANCD2 foci formation.

In addition to playing a role in inducing FANCD2-monoubiquitination, FANCM is also required for inducing replication protein A (RPA) phosphorylation and Chk1 activation in response to ICL-inducing agents [23, 24]. Consistent with this finding, expressing miR146a led to reduced phosphorylation of Chk1 and RPA, which was reversed by FANCM cDNA or anti-miR146a expression (Figure 3E and 3F, respectively). These results confirm that miR146a expression influences the physiological functions of FANCM during DDR.

### miR146a attenuates replication fork restart

**Figure 4:**
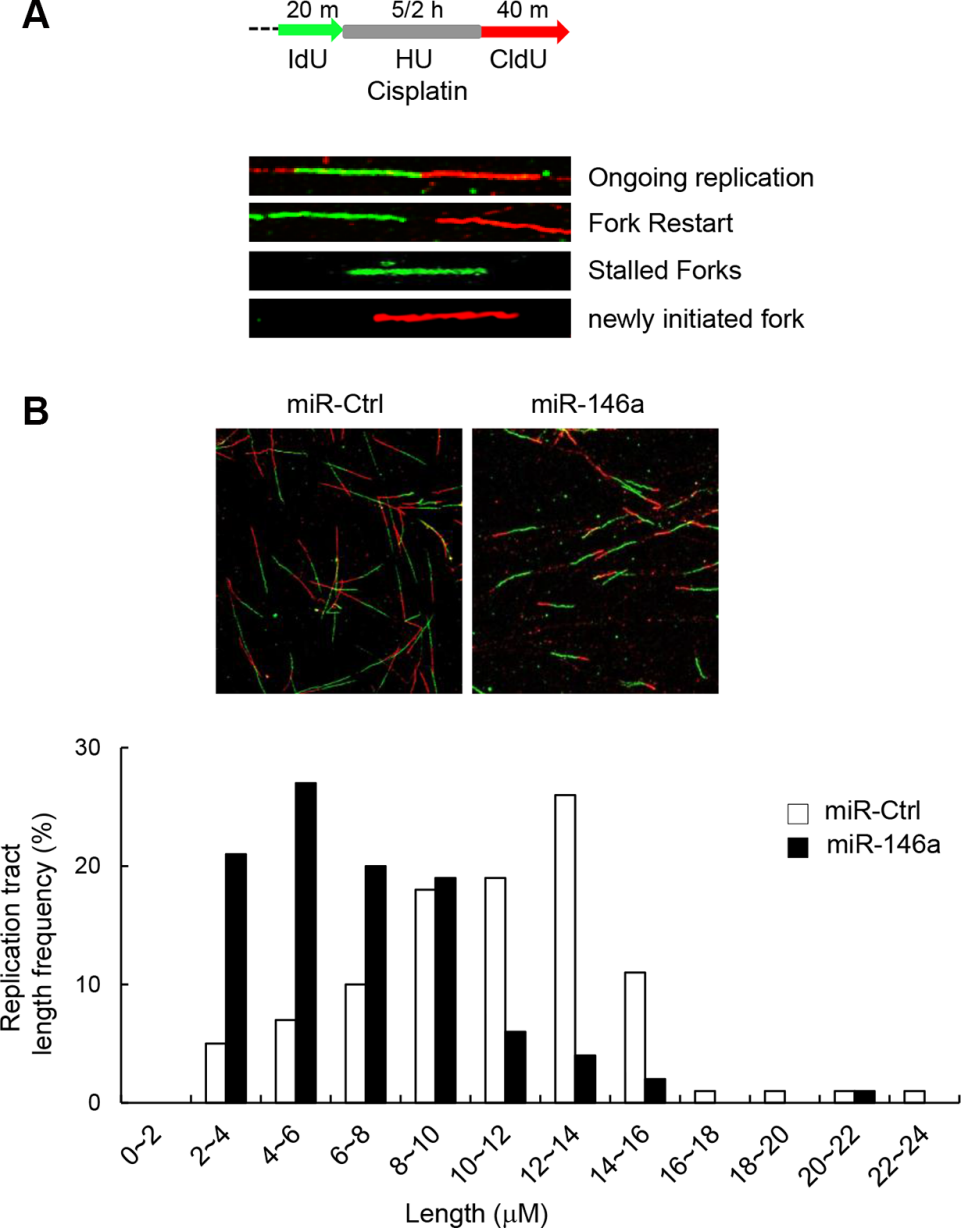
Effect of miR146a on replication fork restart (**A**) Schematic of a single DNA fiber analysis is depicted. For DNA fiber analysis, after being transfected with miR146a or a control miRNA, GES-1 cells were labeled with IdU for 20 min, followed by exposure to HU for 5 hr and chased with CldU for 40 min. (**B**) Representative images of spread DNA fibers from miR146a-expressing cells versus control cells (top). Nascent track length distribution curves were plotted after measuring the length of CldU tracts from DNA fibers with two consecutive colors of green (IdU) and red (CldU).

Given the known roles of FA proteins in modulating replication fork recovery and DNA replication origin firing, we determined the effects of miR146a on replication fork restart. To this end, we analyzed the DNA fiber spreading patterns of miR146a-transfected GES-1 cells after pulse-labeling the cells sequentially with IdU (green fluorescence) and CldU (red fluorescence) before and after 5-h HU treatment, respectively (Figure 4A). The results showed that nascent CldU tract length distribution from stalled replication forks was mainly shorter tracts in miR146a-expressing cells compared to control miRNA-transfected cells (Figure 4B), indicative of attenuated replication fork restart. This miR146a effect is consistent with a previous study demonstrated that FANCM-deficient cells exhibited defective replication fork restart [30].

### Activation of NF-κB recapitulates miR146a expression in suppressing the FA pathway

**Figure 5:**
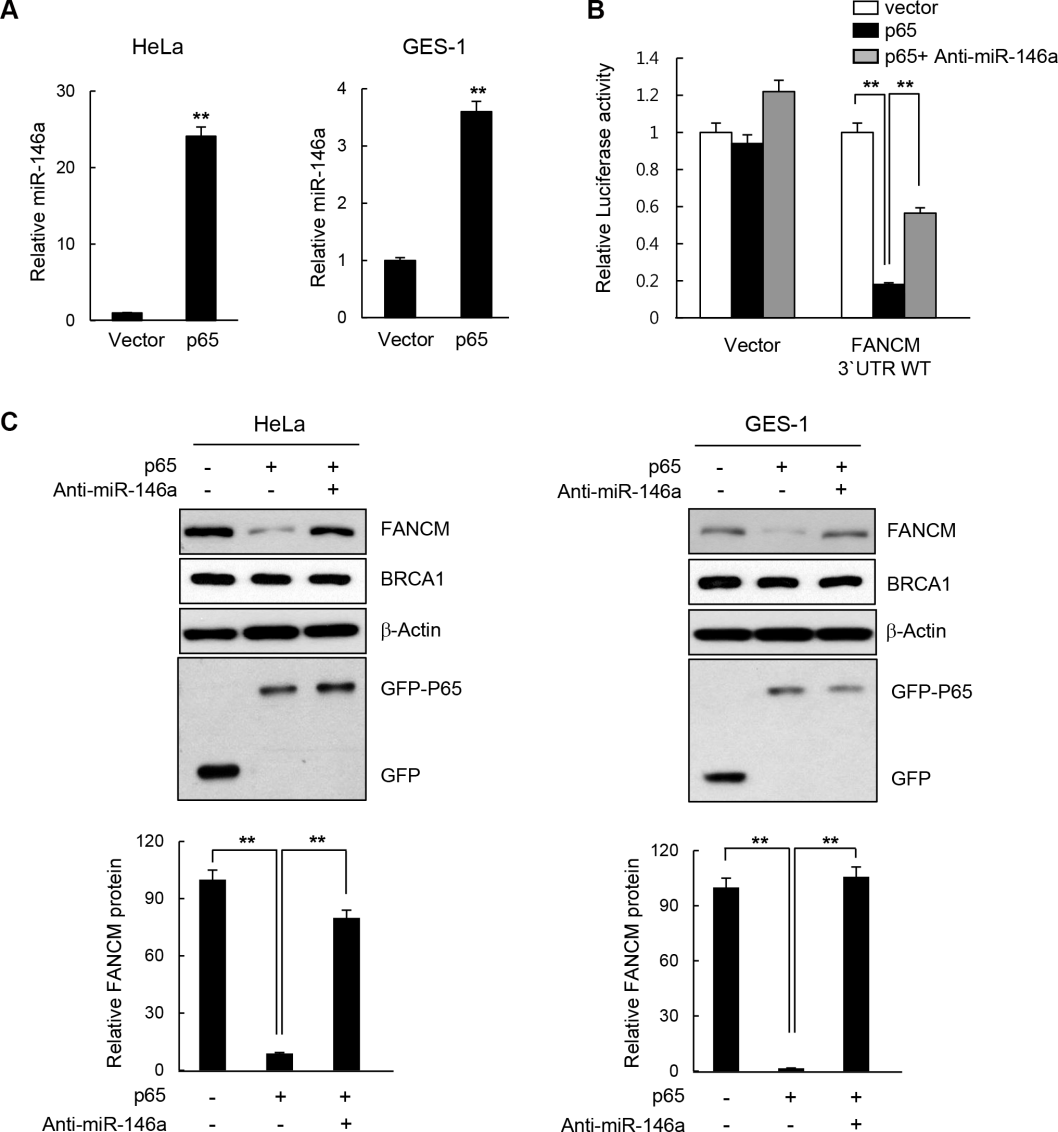
Effect of NF-κB on FANCM expression (**A**) HeLa or GES-1 cells were transfected with the p65 expression construct and miR146a levels in the indicated cells were determined using RT-qPCR analysis. Results are shown as the mean ± SD (*n* = 3); **P < 0.01. (**B**) Luciferase reporter gene construct carrying the wild type or mutated 3′-UTR of FANCM was cotransfected with p65 cDNA or p65 cDNA plus anti-miR146a, as well as a control vector into HeLa cells. Luciferase activity was measured 24 hours after the transfection. Data represent the mean ± SD (*n* = 3); **P < 0.01. (**C**) HeLa or GES-1 cells were transfected with p65 construct in a combination of Anti-miR146a or a control miRNA, and indicated protein levels were determined by western blotting (top). Quantitative densitometry of FANCM protein expression is shown below, expressed as the mean ± SD (*n* = 3).

**Figure 6:**
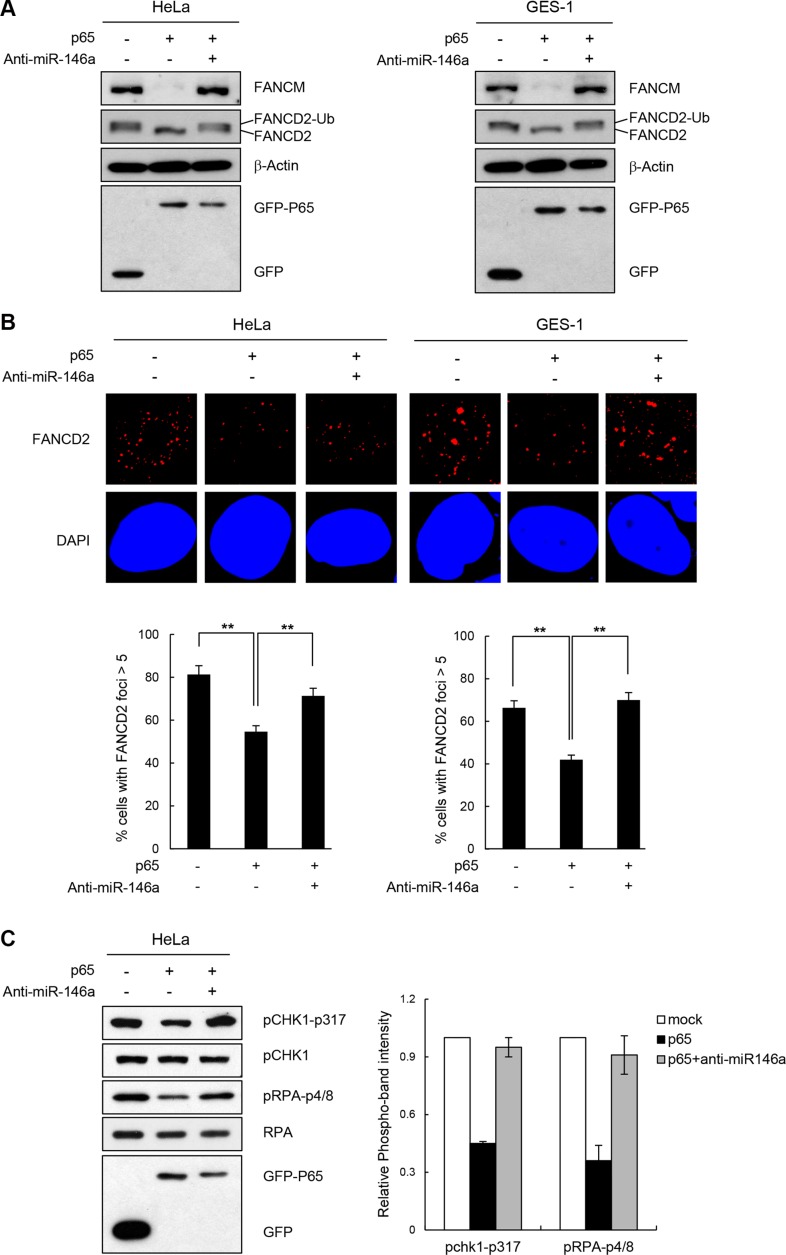

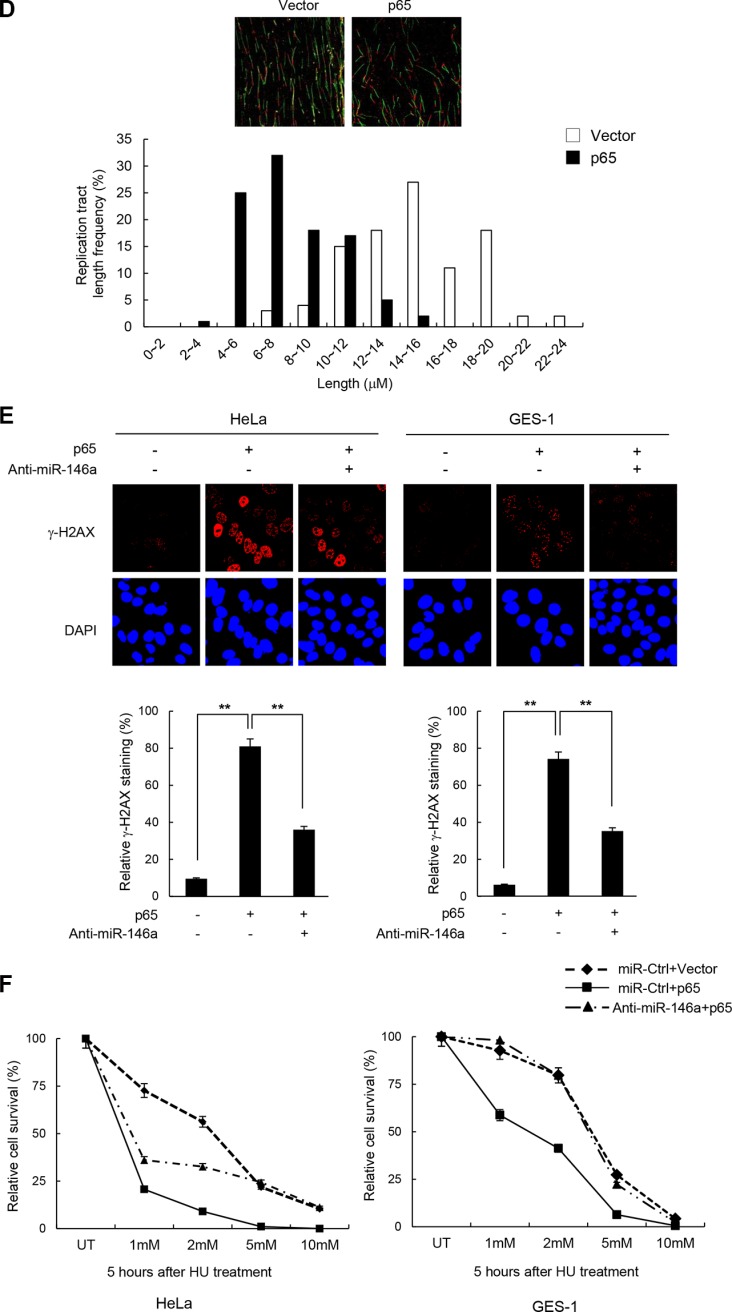
NF-κB reiterating miR146a function in the FA pathway and replication fork restart (**A**) Two days after transfection of cells with p65 cDNA and anti-miR146a in combination as indicated, cells were treated with 5 mM HU for 5 h. The levels of FANCM and p63 proteins, and FANCD2 ubiquitination were measured by western blotting. (**B**) After undergoing the same treatment as (A) HeLa cells were analyzed for FANCD2 foci formation. Results are shown as the mean ± SD (*n* = 3); **P < 0.01. (**C**) Under the same conditions described in (A) cell lysates were analyzed by western blotting with antibodies against pCHK1-S317, CHK1, pRPA-S4/8, RPA, and GFP. Right, quantitation of pCHK1-S317 and pRPA-S4/8 expression. Results are shown as the mean ± SD (*n* = 3). (**D**) GES-1 cells transfected with p65 construct were applied to DNA fiber analysis. Resulting images of DNA fibers of p65-overexpressing cells compared with control cells (top). CldU tract length distribution was determined (bottom). (**E**) HeLa or GES-1 cells with the same treatment as A were subjected to γ-H2AX detection. Results are shown as the mean ± SD (*n* = 3); **P < 0.01. (**F**) HeLa and GES-1 cells were transfected with p65 construct and Anti-miR146a in combinations as indicated and were treated with different doses of HU for 5 hr. Cell survival thereafter was measured using clonogenic survival assay. Results are shown as the mean ± SD (*n* = 3); **P < 0.01.

There is a wealth of evidence supporting the fact that NF-κB up-regulates miR146a expression in various cell types such as those involved in immune responses and in cancer development [4–6]. To test whether the effects of miR146 overexpression described above could be recapitulated by NF-κB activation, we analyzed the effects of overexpressing the NF-κB RelA/p65 subunit after transfecting HeLa or GES-1 cells with the RelA/p65 expression construct. First, RT-qPCR confirmed that the overexpressed p65 subunit significantly upregulated miR146a expression compared to the control vector, indicating that overexpressed RelA/p65 was successfully integrated into the active dimeric form of NF-κB to transactivate miR146a transcription (Figure 5A). NF-κB activation in HeLa cells through p65 overexpression was also confirmed by the NF-κB luciferase assay, where transcription of the *firefly* luciferease was turned on by the binding of activated NF-κB to its *cis*-acting element in the promoter of this reporter gene (Supplementary Figure S7). These results suggest that overexpressing p65 functionally mimics NF-κB activation. Indeed, like miR146a, p65 overexpression in HeLa cells inhibited expression of the luciferase reporter gene carrying wild type FANCM 3′-UTR, whereas co-transfection with the p65 construct and anti-miR146a alleviated this inhibitory effects of NF-κB (Figure 5B), indicating that the effects of NF-κB on FANCM are exerted via miR146a. Western blot analysis showed a prominent reduction in FANCM protein levels, upon overexpression of the p65 subunit, which was recovered by co-transfection of p65 and anti-miR146a (Figure 5C). Consistent with the reduced FANCM levels, overexpressing p65 led to a reduction in FANCD2 monoubiquitination (Figure 6A) and foci formation (Figure 6B) in HeLa and GES-1 cells. These phenotypes were reversed by co-transfection with anti-miR146a, indicating that the effect of NF-κB on FANCD2 monoubiquitination and foci formation rely on the regulatory axis of NF-κB-miR146a-FANCM. Consistent with the role of FANCM in the checkpoint response, p65 overexpression led to decreased Chk1 (Ser317) and RPA phosphorylation, which was reversed by an antagomir of miR146a (Figure 6C). Similar to what we observed in miR146a-transfected cells, p65 overexpression led to delayed recovery from a stalled replication fork, as demonstrated by the shortening of red fluorescent CldU tracts in p65-overexpressing cells compared to control cells (Figure 6D).

We found that p65 overexpression strongly induced γ-H2AX foci, and interestingly, the co-expression of anti-miR146a reduced the phenotype, in HeLa and GES-1 cells (Figure 6E). Consistent with the increased damage, p65-overexpressing cells were sensitive to HU treatment, and the effects were partially or largely alleviated by anti-miR146a treatment (Figure 6F). These data suggest that aberrantly overexpressed NF-κB promotes genomic instability by up-regulating miR146a, which suppresses FANCM expression.

### *H. pylori* mediates down-regulation of FANCM

**Figure 7:**
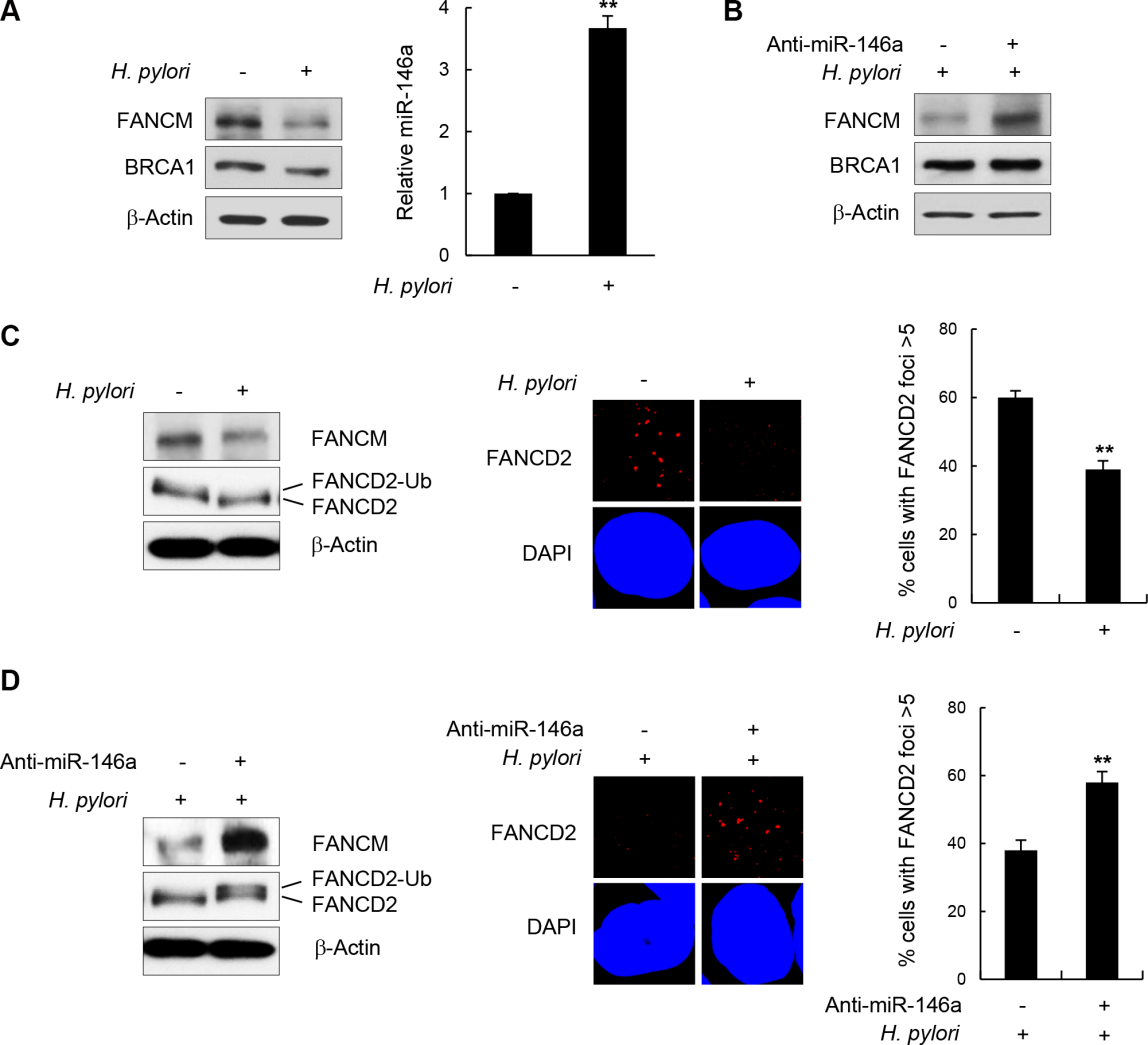
*H. pylori*-mediated FANCM regulation (**A**) After infection of GES-1 cells with *H. pylori*, FANCM and BRCA1 protein levels were determined by western blotting (*left*). The effect of *H. pylori* on miR146a was determined by RT-qPCR (*right*). Results are shown as the mean ± SD (*n* = 3); **P < 0.01. (**B**) GES-1 cells infected with *H. pylori* were transfected with anti-miR146a, and FANCM and BRCA1 protein levels were determined by western blotting. (**C**) *H. pylori* -infected GES-1 cells were treated with 5 mM HU for 5 hours, FANCD2 monoubiquitination (*left*) and its foci forming activity (*right*) were examined by western blotting and immunostaining, respectively. Data respresent the mean ± SD (*n* = 3). **P < 0.01.(**D**) *H. pylori*-infected GES-1 cells were transfected with anti-miR146a and treated with 5 mM HU for 5 hours. FANCD2 monoubiquitinating activity (*left*) and subsequent foci formation (*right*) were measured by western blotting and fluorescent cell staining, respectively. Results are shown as the mean ± SD (*n* = 3). **P < 0.01.

NF-κB is constitutively activated during inflammatory responses triggered by *H. pylori* infection [7–9, 31]. In addition, miR146a was recently identified as a target gene upregulated by *H. pylori* infection [32, 33]. Therefore we postulated that *H. pylori* infection may affect FANCM protein levels by upregulating NF-κB and miR146a in the GES-1 cells. First, to confirm that the inflammatory responses in GES-1 cells were induced by *H. pylori* infection, we assessed the cellular level of the interleukin (IL)-8 transcript using RT-qPCR and verified that its cellular level increased by ~80-folds after *H. pylori* infection compared to an untreated control (Supplementary Figure S8). IL-8 is one of the downstream genes whose expression is turned on by activated NF-κB when *H. pylori* infects gastric mucosa epithelial cells [34–36]. Next, to determine the cellular FANCM level, GES-1 cell lysates were analyzed by western blotting after 24 h of *H. pylori* infection. Consistent with the results from p65 overexpression in Figure 5C, FANCM protein levels were notably down-regulated in *H. pylori*-infected cells compared to untreated control cells (Figure 7A, left). Parallel RT-PCR analysis revealed that miR146a was up-regulated by *H. pylori* infection by ~3.5 fold compared to the control (Figure 7A, right). Importantly, the effects of *H. pylori* infection on FANCM expression were alleviated by the simultaneous treatment of anti-miR146a (Figure 7B), suggesting that these effects were largely due to miR146a expression.

Consistent with the reduction in FANCM levels, FANCD2 monoubiquitination and foci formation were reduced upon *H. pylori* infection (Figure 7C). In contrast, the effects of *H. pylori* on FANCD2 were largely reversed by transfecting anti-miR146a during *H. pylori* infection, as shown by recovery of both FANCD2 monoubiquitination and foci formation (Figure 7D). These findings indicate that *H. pylori*-induced inflammatory responses down-regulate FANCM expression by increasing cellular miR146a, whose expression is under the control of NF-κB.

## DISCUSSION

In this study, we demonstrated that FANCM is a direct target of miR146a. Through bioinformatic searches, we identified the 3′UTR of FANCM as a binding site of miR146a, and confirmed this experimentally. Overexpression of miR146a suppressed the steady state level of FANCM, which was reversed by an antagomir. We showed that miR146a overexpression disrupted the several known FANCM-dependent processes, such as: FANCD2 monoubiquitination/foci formation, the FANCM-mediated checkpoint response, replication fork stability, and cellular resistance to cisplatin and HU. miR146a overexpression also led to the reduced RAD51 foci formation and increased double-strand break formation, although these phenotypes could be attributed to both impaired FANCD2 activation as well as BRCA1 expression. BRCA1 was previously reported to be a target of miR146a in a study which showed that miR146a overexpression reduced HR repair by BRCA1 downregulation [27]. Thus, we used BRCA1 as an internal control throughout our studies. Interestingly, we found that the effects of miR146a were recapitulated by overexpressing the p65 subunit of NF-κB, and that these effects were significantly reversed by the concomitant expression of anti-miR146a, suggesting that the effects caused by the NF-κB activation are largely due to miR146a. In order to utilize a more physiological inducer of miR146a expression, we employed *H. pylori* infection as a tool. Indeed, *H. pylori* led to a significant induction of miR146a, as well as reduced levels of FANCM and FANCD2 activation, which were reversed by anti-miR146a. Thus, our findings show that genome integrity is influenced by the functional link among pathogen-induced inflammation, NF-κB, and miR146a.

In light of the fact that implication of miR146a in tumorigenesis has been heavily discussed [5, 6, 26, 27, 37], our results further strengthen the notion that the aberrant expression of miR146a can increase tumorigenesis. Our finding that the breast cancer cell lines expressing a high level of miR146a failed to express the FANCM protein is particularly intriguing. Our work shows that the downregulation of FANCM at least in part contributes to the role of miR146a in compromising genome integrity. Although the statistically significant inverse correlation between miR146a and FANCM needs more extensive comparative studies in various types of cancer cells with clinical manifestation, our study provides the first line of evidence showing a negative correlation between endogenous miR146a and FANCM expression in breast cancer cell lines.

Our analysis suggests that the miR146a-mediated regulation of FANCM may be restricted to primates, based on the poorly conserved sequence within the FANCM 3′UTR. This finding suggests that sequences corresponding to the FANCM 3′UTR might have undergone massive species-specific gene rearrangements such as insertions and deletions and base substitutions after speciation since early vertebrate evolution [38]. Moreover, the alignment of the region spanning two miR146a target sites and their flanking regions were not applicable except in primates, nor were two target sites detected in the other species than primates through a BLAST search. This indicates that a regulation strategy for FANCM via miR146a has emerged as a relatively recent gain-of-function in the evolutionary history of vertebrate FANCM. As per the regulation of the FA pathway by miRNA, a several other FA-related genes such as BRCA1 and RAD51 were shown to be targeted by miRNAs [39–41].

Perhaps the most intriguing aspect of our work is the connection between pathogenic infection and disruption of genome integrity. Pathogens such as *H. pylori* have long been suspected as a potential carcinogen. Individuals afflicted with chronic infection of this microbe are at high risk for gastric cancer or gastric mucosa-associated lymphoid tissue lymphoma [42–44]. We found that miR146a expression was up-regulated by both *H. pylori* infection and NF-κB activation, the latter of which is regarded as a critical link between inflammation and cancer. While the contribution of inflammatory cytokines to inflammation-linked cancer susceptibility is now widely accepted, the DNA damage accompanying chronic inflammation has gradually gained attention as it can lead to gene mutations that can cause cancer [28, 45]. During inflammation, pro-inflammatory cytokines such as TNF-α, IL-1β, and IL-6, serve as the main way of communication between cells in different tissues and immune cells and trigger intracellular signaling cascades whose main feature is NF-κB activation [46–49]. miR146a is a well-known NF-κB-dependent gene but has also been reported to downregulate several signaling mediators involved in inflammatory responses such as TNF receptor-associated factor 6 (TRAF6) and IL-1 receptor-associated kinase 1 (IRAK1) that are located upstream of NF-κB activation, thus exerting a negative feedback loop for modulating NF-κB activity [4, 50, 51], which prevents constitutive activation of NF-κB during inflammatory response. On the other hand, NF-κB can be activated during the host cell response to bacterial infection (e.g., *H. pylori* in our study). *H. pylori*'s cell wall component peptidoglycan which is injected into gastric epithelial cells during infection, is recognized by the intracellular pattern recognition molecule, NOD1, eliciting the signaling cascade to activate NF-κB, which occurs independently of cytokine-triggered signaling cascades [7, 33, 52]. Our results demonstrate that GES-1 cells successfully responded to *H. pylori* infection, expressing IL-8 whose transcription is activated by NF-κB. Although additional studies on *H. pylori*-induced dampening of FA pathway activation in more physiological settings are needed, our finding that *H. pylori* influenced FANCM levels provides an understanding of the mechanism behind *H. pylori*-linked carcinogenesis. There are several ways to explain the mechanisms underlying genomic instability caused by *H. pylori*: i) *H. pylori* infection impairs mismatch repair and base excision repair by down-regulating component molecules [53, 54]; ii) *H. pylori* infection generates reactive oxygen species, causing oxidative damage of the DNA and genomic instability [55]; iii) specific oncogene activation in precancerous patient tissue samples is associated with double-strand breaks caused by *H. pylori* infection and the activation of DNA-damage checkpoints [56–58]. The disturbance of the FA pathway by *H. pylori* may not be critical for maintenance of the genomic integrity of terminally-differentiated gastric epithelial cells in the G_0_ phase. However, this pathogen would compromise the genomic integrity if the infection occurs in actively proliferating gastric epithelial progenitors [59]. Subsequent exposure to genotoxins such as ICL-inducing agents, which gastric epithelial cells have even higher chance to take up as food ingredients may also foster the gastric cancer development.

In summary, we demonstrated that miR146a decreased the FANCM protein level, thereby disabling FANCD2 activation upon DNA damaging agent treatment. Furthermore, we uncovered an important role of miR146a in NF-κB- and H. pylori- induced genomic instability. Our work suggests that dysregulated NF-κB activation and persistent *H. pylori* infection, both of which upregulate miR146a expression, can compromise genome integrity and potentially contribute to tumorigenesis in part by suppressing the DDR involving FANCM.

## MATERIALS AND METHODS

### Cell culture and treatment

The human cervical adenocarcinoma cell line HeLa, immortalized human normal gastric epithelial mucosa cell line GES-1, human gastric carcinoma cell line HGC-27, and human osteosarcoma bone morphogenetic cell line U2OS were purchased from the American Type Culture Collection (ATCC, Rockville, MD) and maintained as recommended: all of the cell types were cultured in Dulbecco's Modified Eagle's medium (Invitrogen, CA) supplemented with 10 % heat-inactivated fetal bovine serum (Invitrogen), 100 units/ml penicillin, and 100 μg/ml streptomycin sulfate (Invitrogen), and maintained in a humidified incubator containing 5% CO_2_ at 37°C. To induce replication fork stalling or ICL damage, exponentially growing cells were exposed to 5 mM HU or 1 mM cisplatin and allowed to recover at 37°C for 5 h.

### *H. pylori* culture and infection

The *H. pylori* strain 26695 was obtained from HpKTCC (*H. pylori* Korean Type Culture Collection, Jinju, South Korea). The bacteria was grown in Brucella broth containing 10% fetal bovine serum, 10 μg/ml vancomycin, 25 μg/ml nalidixic acid and 5 μg/ml amphotericinB, and maintained in a humidified incubator containing 10% CO_2_ at 37°C. GES-1 cells were seeded at 10 × 10^5^ cells/100 mm plate and grown to 70–80% confluency. *H. pyroli* infected the GES-1 cells at 100 MOI multiplicity of infection for 24 h. The infection was monitored by the release of IL-8.

### Antibodies

The antibodies used for Western blotting were as follows: FANCM (sc-98710), FANCD2 (sc-20022), BRCA1 (sc-642), β-actin (sc-47778; Santa Cruz, CA), RPA (NA18; Calbiochem, CA), phosphor-RPA(S4/S8) (A300-245A; Bethyl Lab), CHK1 (2G1D5; Cell Signaling, MA) and phosphor-CHK1(S317) (AF2054; R&D System, MN). FANCD2 foci were detected by immunofluorescence staining using an anti-FANCD2 antibody (AB2187; Abcam). γ-H2AX foci were visualized by immunofluorescence staining using anti-γ-H2AX mouse monoclonal antibody (JBW301; Upstate Biotechnology, NY). RAD51 foci were detected by immunofluorescence staining using mouse monoclonal anti-RAD51 (14B4) (ab213; Abcam, MA).

### miRNA luciferase reporter assay

Segments of the 3′UTR of FANCM containing putative miR146a binding sites were cloned into pMIR-REPORT *firefly* luciferase vector (Applied Biosystems, CA). Deletion mutants of predicted miR146a binding sites were made using the GENEART Site-Directed Mutagenesis kit (Invitrogen). For the luciferase activity assay, pMIR-REPORT luciferase vectors containing wild type or mutant 3′UTRs of FANCM and pRL-TK vector containing *Renilla* luciferase as a transfection control were co-transfected into HeLa cells using Lipofectamine 2000 (Invitrogen), and subsequently, the same cells were transfected with miR146a, anti-miR146a or RelA/p65 expression construct. After 24 h of transfection, the luciferase assay was performed using the dual luciferase reporter assay system (Promega, WI) according to the manufacturer's instructions. Luciferase activity was quantified using a luminometer (Glomax, Promega).

### NF-κB luciferase reporter assay

HeLa cells were seeded at a concentration of 5 × 10^5^ cells/well in 24-well plates. After overnight culture and reaching 80% confluence, the cells in each well were transiently transfected with 1 μg DNA consisting of pGL4.32 (luc2P/NF-κB-RE/Hygro) as a reporter (Promega), pRL-TK, as well as the RelA/p65 expression construct, in serum-free medium according to the manufacturer's protocol. After 12 h, the transfection mix was removed and replaced with complete medium. Twenty-four hours after co-transfection, cells were harvested and luciferase activity was determined using the Luciferase Assay System (Promega, E1500).

### miRNA and plasmid transfection

Hsa-miR146a duplex and negative control miRNA were purchased from Bioneer (Daejeon, South Korea). Cells were transfected with 50 nM miRNA using lipofectamine RNAiMax (Invitrogen) according to the manufacturer's instructions. For rescue experiments, a miR146a inhibitor (anti-miR146a) was used. Full-length BRCA1 and FANCAM were cloned into pcDNA3-HA and pCMV-Flag vector, respectively, to generate mammalian expression vector. For experiments measuring NF-κB-mediated miR146a regulation, pcDNA HA-p65 vector was used. pcDNA-HA empty vector and scrambled oligonucleotide were used as negative controls.

### Western blotting

Cells were lysed in RIPA buffer (50 mM Tris-HCl [pH 7.5], 150 mM NaCl, 1% Nonidet P-40, 0.5% sodium deoxycholate, 0.1% sodium dodecyl sulfate, 1 mM dithiothreitol, 1 mM phenylmethanesulfonyl fluoride, 10 μg/ml leupeptin and 10 μg/ml aprotinin). Equal amounts of cell or tissue extracts were separated by 6–12% SDS-PAGE gels followed by electrotransfer onto a PVDF membrane (Life Sciences, NJ). Western blots were performed by using the appropriate primary and secondary antibodies. The amounts of FANCM protein were quantified using Scion Image software (Scion Corp., MD).

### Immunofluorescence staining

Cells cultured on cover slips were treated with 5 mM hydroxyurea or 1 mM cisplatin, allowed to recover for adequate times and then fixed in 4% paraformaldehyde and 98% methanol, followed by permeabilization with 0.3% Triton X-100. Then coverslips were blocked in 5% BSA in PBS and immunostained with primary antibodies and secondary antibodies conjugated with Alexa Fluor 488 (green, Molecular Probe, CA) or Alexa Fluor 594 (red, Molecular Probe). After washing, the coverslips were mounted onto slides using a Vectashield mounting medium with DAPI (Vector Laboratories, CV). Fluorescence images were taken using a confocal microscope (Zeiss LSM 510 Meta; Carl Zeiss) and analyzed with Zeiss microscopic imaging software ZEN (Carl Zeiss).

### Array comparative genomic hybridization analysis

Array comparative genomic hybridization (CGH) analysis was performed using the Nimblegen Human CGH 12 × 135 K whole-genome tiling v3.1 Array (Agilent Technologies, CA). Human genomic DNA (1 μg) from human fibroblast GM00637 cells which were transfected with miR146a and reference DNA samples from control cells were independently labeled with fluorescent dyes (Cy3/Cy5), co-hybridized at 65°C for 24 h, and then subjected to the array. The hybridized array was scanned using NimbleGen MS200 scanner (NimbleGen Systems Inc., WI) with 2 μm resolution. Log_2_-ratio values of the probe signal intensities were calculated and plotted versus genomic position using Roche NimbleGen NimbleScan v2.5 software. Data are displayed and analyzed in Roche NimbleGen SignalMap software and CGH-explorer v2.55.

### DNA fiber analysis

GES-1 cells were labeled with 25 μM 5-iodo-2′-deoxyuridine (IdU) for 20 min, followed by exposure to 5 mM HU for 5 h, and chased with 250 μM 5-chloro-2′-deoxyuridine (CldU) for 40 min. After labeled cells were harvested, DNA fibers were spread as previously described [60] before fluorescent staining for IdU and CldU tracts (with primary antibodies for IdU, (mouse α-BrdU (Sigma) at 1:25 dilution); and for CldU (rat α-BrdU (Sigma) at a 1:400 dilution) and secondary antibodies: anti-mouse IgG conjugated with AlexaFluor 488 and Cy3-conjugated anti-rat IgG, respectively, from Invitrogen). Fibers were visualized under a confocal microscope (Zeiss LSM 510 Meta) and analyzed using Zeiss microscopic imaging software ZEN (Carl Zeiss).

### Clonogenic cell survival assay

After treatment of HU or cisplatin at different concentrations for 2 h, 5 ×10^2^ cells were immediately seeded on 60-mm dishes in triplicate and grown for 2~3 weeks at 37°C to allow colonies to form. Colonies were stained with 2% methylene blue/50% ethanol and were counted. The fraction of surviving cells was calculated as the ratio of the number of colonies to the cell number at the time of seeding for the plating efficiency of treated cells over untreated cells. Cell survival results are reported as the mean value ± SD for three independent experiments.

### RNA extraction and reverse rranscription-quantitative real-time PCR (RT-qPCR)

Total RNA was isolated from cultured cells, mouse tissues, and prostate cancer samples using TRIzol reagent (Invitrogen). For quantitation of *FANCM* and *IL-8* mRNA, cDNA was synthesized using 1 μg total RNA, random hexamer (Promega) and M-MLV reverse transcriptase (Invitrogen). Real-time PCR analysis was performed using the SYBR green-based fluorescent method (SYBR premix Ex Taq kit, TaKaRa Bio) and the MX3000P® qRT-PCR system (Stratagene, CA) with specific primers. Primers used for real-time PCR are as follows: *fancm* forward, 5′-tgctcttcacaggagtggtg-3′ and *fancm* reverse, 5′-gggcacacaggaacttgact-3′; *IL-8* forward, 5′-TTGGCAGCCTTCCTGATTTC-3′ and reverse 5′-AACTTCTCCACAACCCTCTG-3′. To quantify miRNAs, cDNA was synthesized using Mir-X^TM^ miRNA first-strand synthesis and SYBR qRT-PCR kit (Clontech) according to the manufacturer's instructions. Has-miR146a-MI0000477 was used as primer for real-time qPCR. The quantity of transcripts was calculated based on the threshold cycle (C_t_) using the delta-delta C_t_ method that measures the relative of a target RNA between two samples by comparing them to a normalization control RNA (*gapdh* or *U6*).

### Statistical analysis

Data in all of the experiments are presented as the mean ± standard deviation (SD) Statistical comparisons were performed using two-tailed paired Student's *t*-test, where *p* < 0.01(**) was considered statistically significant. Analyses were performed using GraphPad Prism software (GraphPad software) and Excel (Microsoft).

## Supplementary Materials


