# Correction: miR146a-mediated targeting of FANCM during inflammation compromises genome integrity

**DOI:** 10.18632/oncotarget.27481

**Published:** 2020-05-26

**Authors:** Devakumar Sundaravinayagam, Hye Rim Kim, TingTing Wu, Hyun Hee Kim, Hyun-Seo Lee, Semo Jun, Jeong-Heon Cha, Younghoon Kee, Ho Jin You, Jung-Hee Lee

**Affiliations:** ^1^ Laboratory of Genomic Instability and Cancer Therapeutics, Cancer Mutation Research Center, Chosun University School of Medicine, Gwangju, Republic of Korea; ^2^ Department of Pharmacology, Chosun University School of Medicine, Gwangju, Republic of Korea; ^3^ Department of Cellular and Molecular Medicine, Chosun University School of Medicine, Gwangju, Republic of Korea; ^4^ Department of Oral Biology, Department of Applied Life Science, The Graduate School, Yonsei University College of Dentistry, Seoul, Republic of Korea; ^5^ Department of Cell Biology, Microbiology, and Molecular Biology, College of Arts and Sciences, University of South Florida, Tampa, Florida, United States of America

**This article has been corrected:** During the assembly of Figure 3E and 3F, the western blot of pCHK1-p317 in Figure 3F was inadvertently used for pRPA-p4/8 in Figure 3E. The corrected Figure 3 is shown below. The authors declare that these corrections do not change the results or conclusions of this paper.

Original article: Oncotarget. 2016; 7:45976–45994. 45976-45994. https://doi.org/10.18632/oncotarget.10275

**Figure 3:**
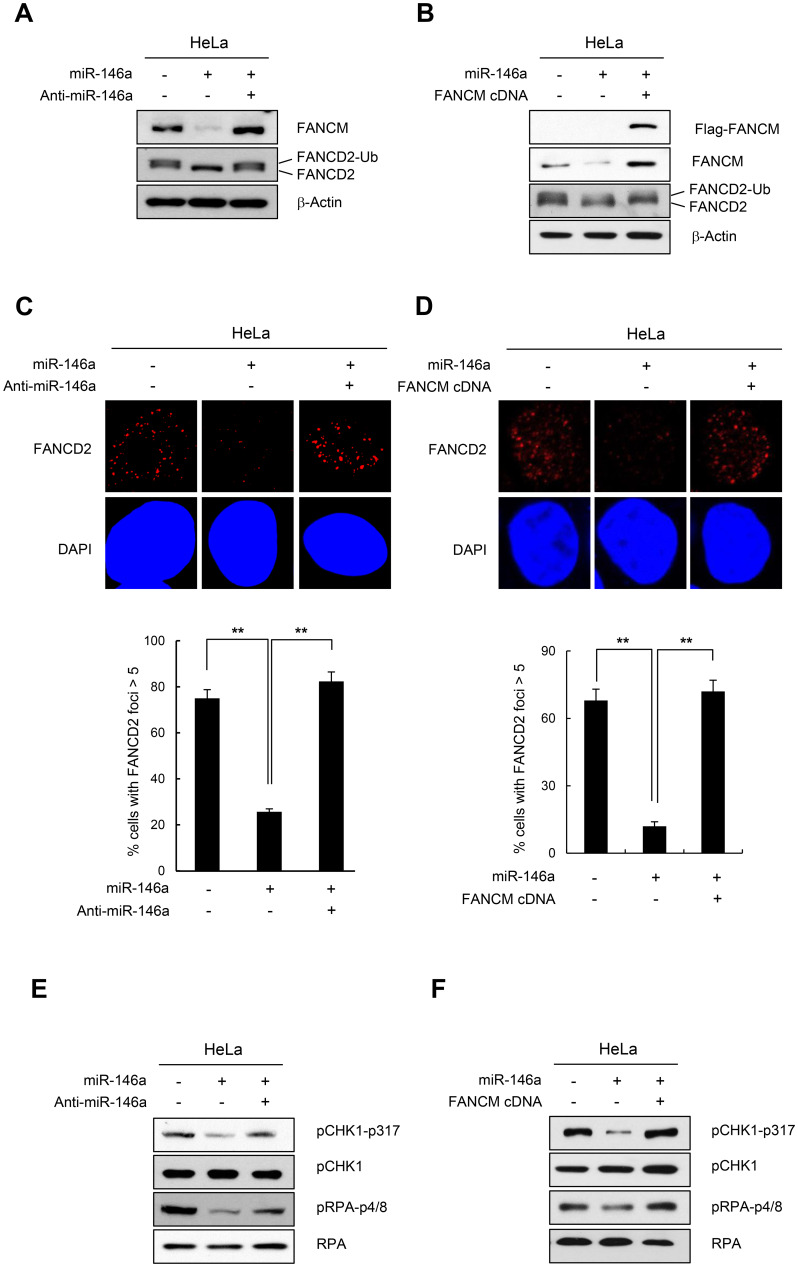
Effect of miR146a on FANCD2 monoubiquitination and the FA pathway. (**A** and **B**) HeLa cells were transfected with miR146a in the absence or presence of anti-miR146a (**A**) or miR146a-insensitive FANCM cDNA (**B**). After a 48h transfection, the cells were treated with 5mM HU for 5 h. The protein levels of FANCM and FANCD2 were measured by western blotting. Monoubiquitinated FANCD2 is indicated as the upper band of doublet protein bands corresponding to FANCD2. (**C** and **D**) After HeLa cells underwent the same treatment as **A** and **B**, cells were analyzed for FANCD2 foci formation. DAPI was used for nuclear staining. Results are shown as the mean ± SD (n = 3); ^**^ *P* < 0.01. (**E** and **F**) Indicated cells were treated with 5mM HU for 5 hr. Cell lysates were analyzed by Western blotting with antibodies against pCHK1-S317, CHK1, pRPA-S4/8, and RPA.

