# Supplementary material for: miR146a-mediated targeting of FANCM during inflammation compromises genome integrity
Source: Oncotarget. 2016 Jun 24;7(29):45976–94. doi: 10.18632/oncotarget.10275 (PMC5216775; doi:10.18632/oncotarget.10275)
Supplement: Supplementary file 1 [file oncotarget-07-45976-s001.pdf]

miR146a-mediated targeting of FANCM during inflammation compromises genome integrity

Supplementary Materials

Supplementary Table S1: miRNAs predicted to bind the 3′ UTR of the FANCM gene by Targetscan

<table>
<tr><th>miRNA
Seed sequence
position of
FANCM 3′UTR</th><th colspan="2">Predicted target site in 3′UTR of the FANCM</th></tr>
<tr><td>miR-146a
704 - 711
755 - 761</td><td>5′...UGCUGUUCUAGUGAUAGUUCUCA...
| | | | | | | |
3′ UUGGGUACCUUAAGUCAAGAGU</td><td>5′...CUCUGUUCACUCUGCAGUUCUCU...
| | | | | | |
3′ UUGGGUACCUUAAGUCAAGAGU</td></tr>
<tr><td>miR-101
319 - 325
539 - 545</td><td>5′...CCCAAAAUUAAAGUUGUACUGUU...
| | | | | | | | | |
3′ AAGUCAAUAGUGUCAUGACAU</td><td>5′...GUGUGUCCCAGUUAA---------GUACUGUC...
| | | | | | | | | | | | |
3′ AAGUCAAUAGUGUCAUGACAU</td></tr>
<tr><td>miR-300
119 - 125</td><td>5′ ...UUUAAAUAUUUUAUAUUGUAUAC...
| | | | | |
3′ UCUCUCUCAGACGGGAACAUAU</td><td></td></tr>
<tr><td>miR-342
459 - 465</td><td>5′ ...GGGUGAUGGGUGGGUUGUGAGAU...
| | | | | |
3′ UGCCCACGCUAAAGACACACUCU</td><td></td></tr>
<tr><td>miR-505
342 - 348</td><td>5′ ...GUGAUAAAUAGUAAAGUUGACAU...
| | | | | |
3′ UCCUUUGGUCGUUCACAACUGC</td><td></td></tr>
<tr><td>miR-539
516-522</td><td>5’ ...UGUUAGCUGGUUACCUUUCUCAA...
| | | | | |
3’ UGUGUGGUUCCUAUUAAAGAGG</td><td></td></tr>
</table>

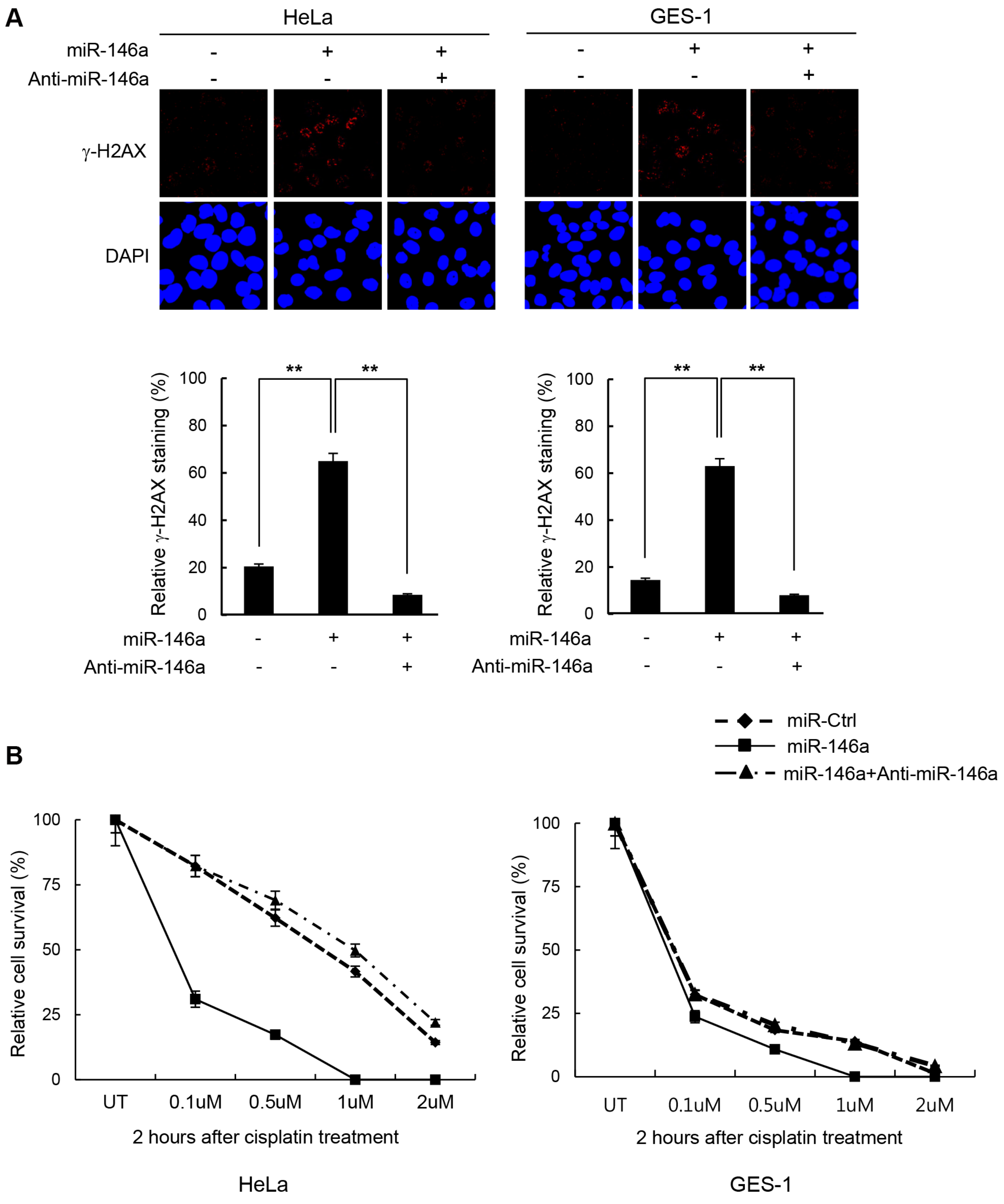

Supplementary Figure S1: Effects of miR146a on recovery from replication stress and cell survival. (**A**) Two days after transfection of HeLa or GES-1 cells with miR146a alone or together with anti-miR146a, the cells were treated with 1 mM cisplatin for 16 h to detect residual γ-H2AX. Data are presented as the mean ± SD ($n = 3$); $^{**}P < 0.01$. (**B**) HeLa and GES-1 cells were transfected with control, miR146a, and miR146a plus anti-miR146a and were then exposed to increasing concentrations of cisplatin for 2 h. The viability of treated cells was examined using the clonogenic survival assay. Results are shown as the mean ± SD ($n = 3$); $^{**}P < 0.01$.

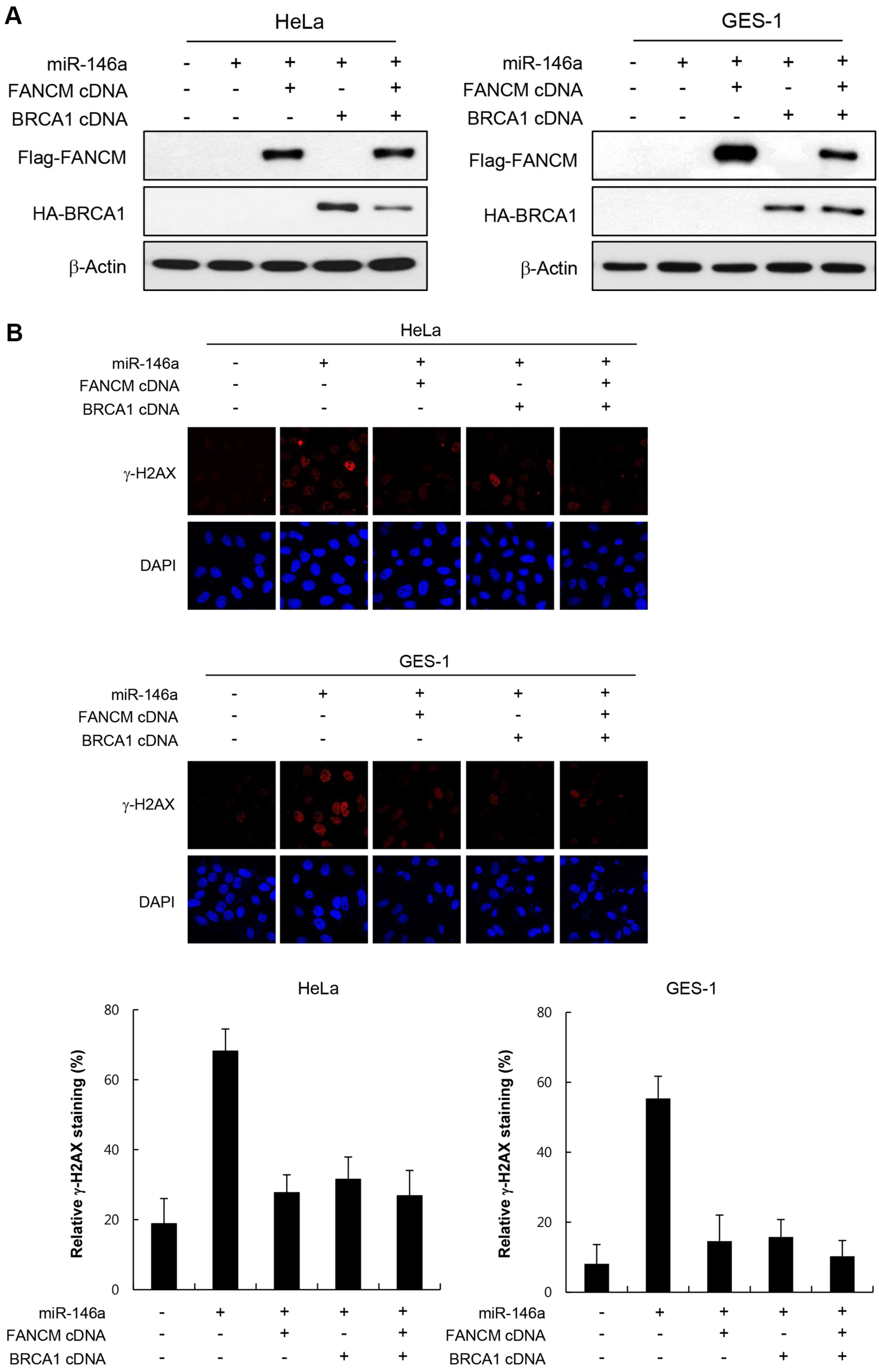
A
HeLa
GES-1
miR-146a
FANCM cDNA
BRCA1 cDNA
Flag-FANCM
HA-BRCA1
β-Actin
B
HeLa
miR-146a
FANCM cDNA
BRCA1 cDNA
γ-H2AX
DAPI
GES-1
miR-146a
FANCM cDNA
BRCA1 cDNA
γ-H2AX
DAPI
HeLa
GES-1
Relative γ-H2AX staining (%)
0
20
40
60
80
miR-146a
FANCM cDNA
BRCA1 cDNA

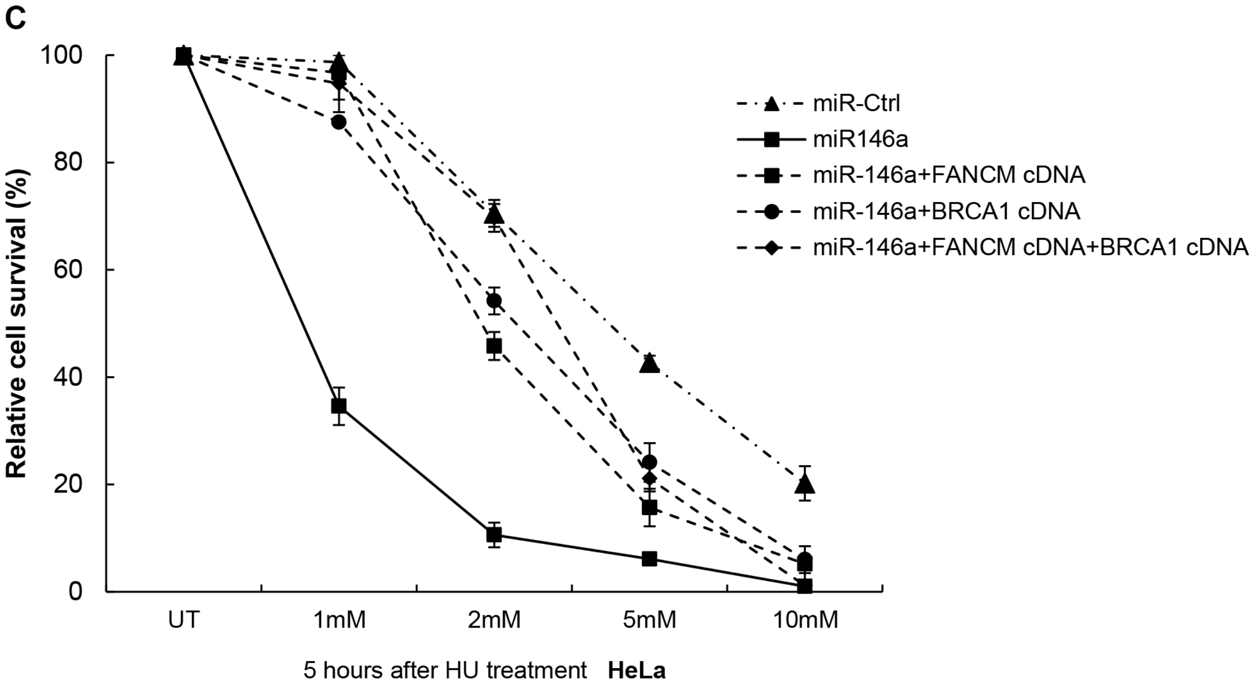

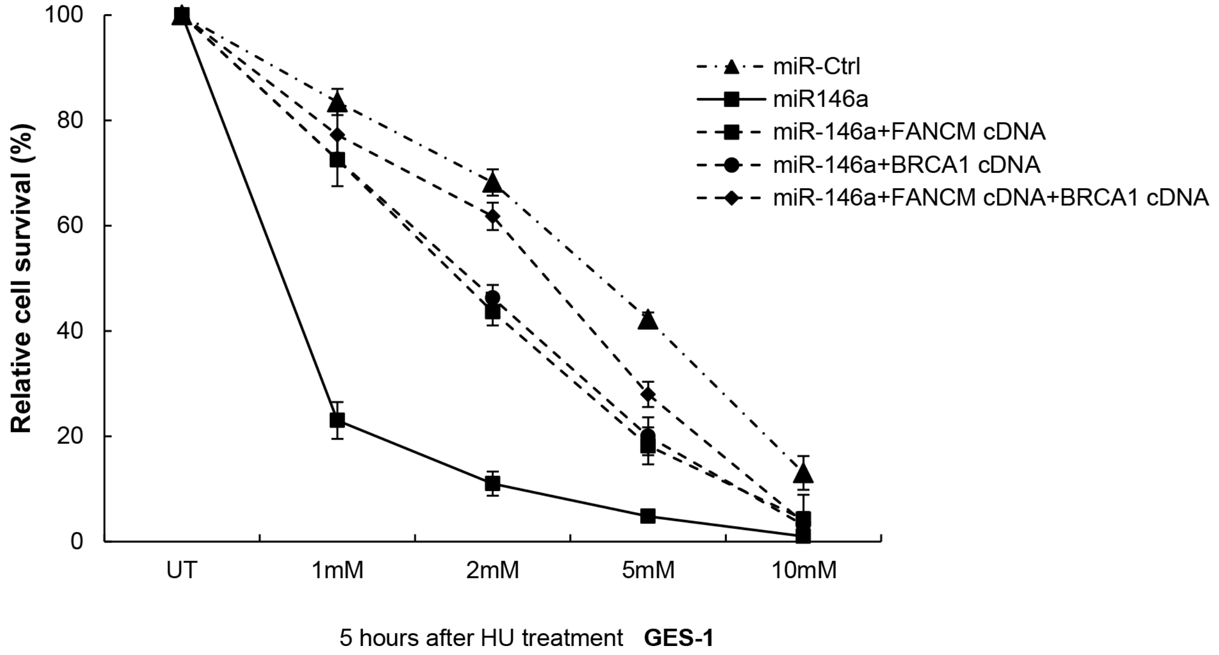

Supplementary Figure S2: miR146a-mediated FANCM and BRCA1 downregulation leads to impaired DNA damage response. (**A**) HeLa or GES-1 cells were transfected with miR146a alone or together with FANCM and/or BRCA1 construct. The levels of indicated proteins were determined using western blotting. (**B**) HeLa and GES-1 cells transfected with indicated combination of miR-146a, FANCM and BRCA1 were treated or not with 5mM HU for 16 h and analyzed residual γ-H2AX foci. Data are presented as the mean ± SD (n = 3); **$P < 0.01$. (**C**) Indicated cells were transfected with microRNA and/or indicated cDNA. The cells were then exposed to increasing concentrations of HU for 5 h. The viability of treated cells was examined using the clonogenic survival assay. Results are shown as the mean ± SD (n = 3); **$P < 0.01$.

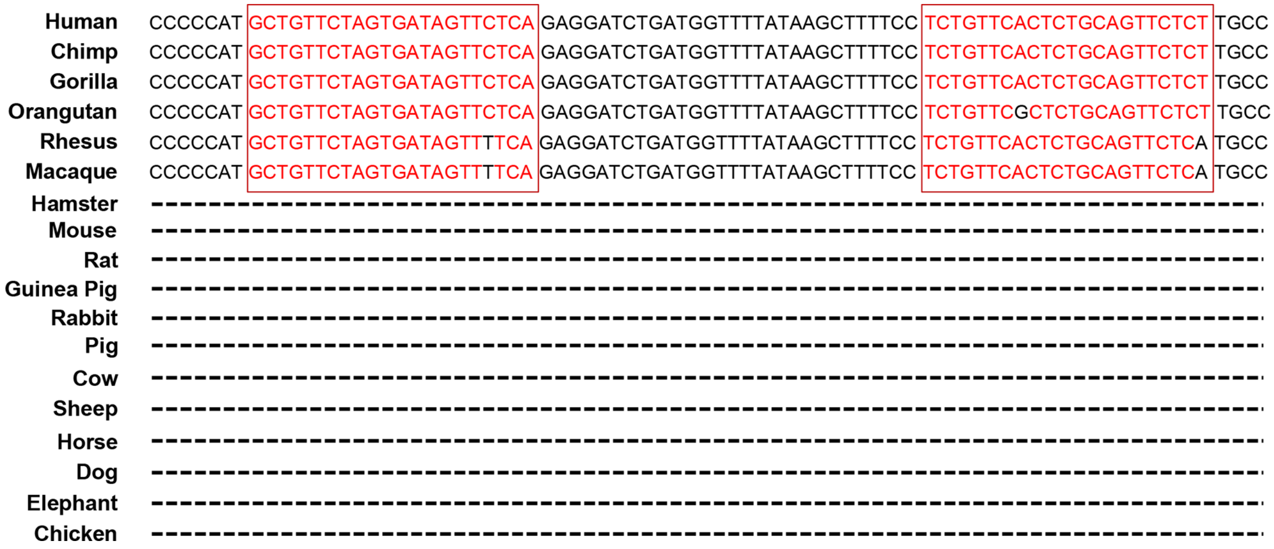

Supplementary Figure S3: Alignment of the human FANCM 3′-UTR with vertebrate species. Part of the human FANCM 3′-UTR containing two miR146a target sites (between nt 697 and 765) were aligned with other vertebrate species as indicated. Two target sites of miR146a are boxed in red and sequence variation in the seed regions of monkey species is presented.

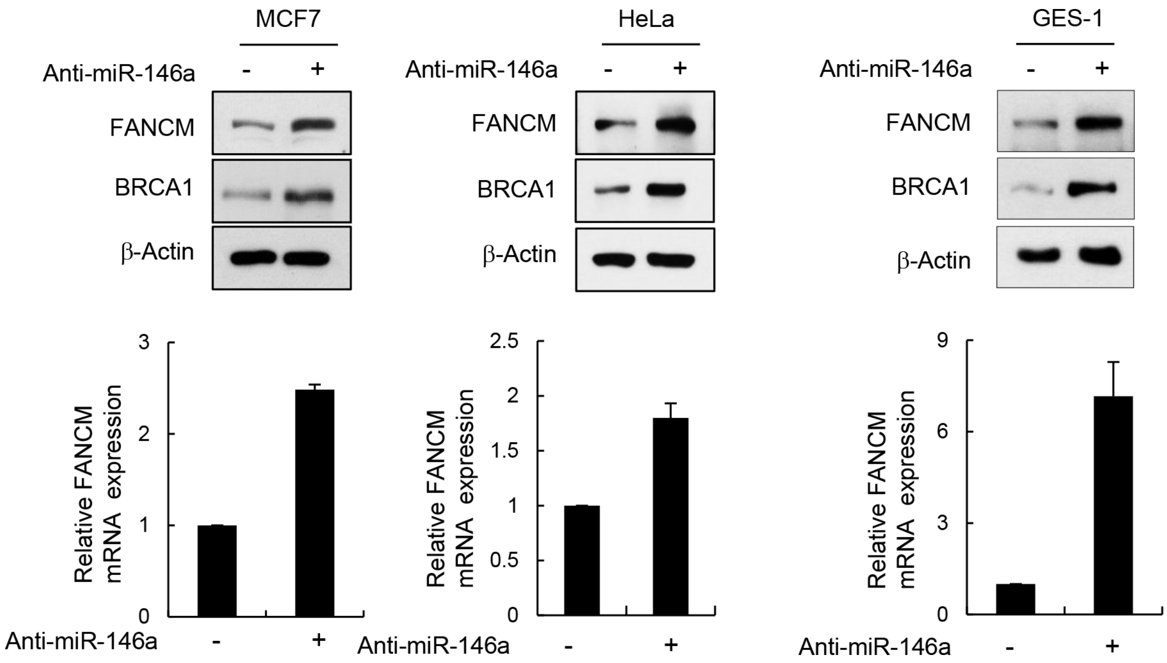

Supplementary Figure S4: Antagonizing miR146a enhances FANCM and BRCA1 expression. MCF7, HeLa and GES-1 cells were transfected with either control anti-miRNA or anti-miR146a, and the level of FANCM and BRCA1 proteins was determined using western blotting.

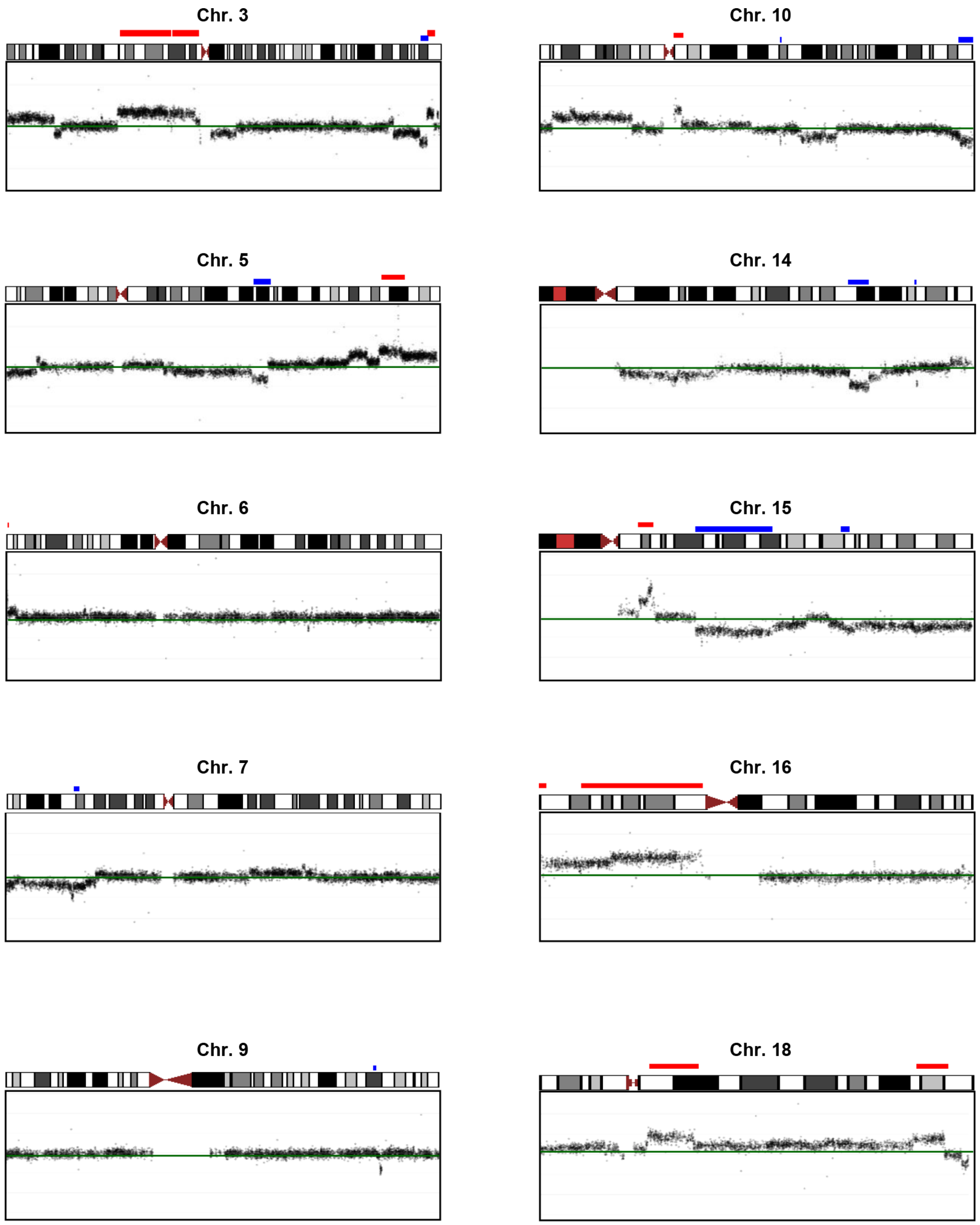

Supplementary Figure S5: Genome-wide chromosomal aberrations due to miR146a overexpression. Using the copy number of control miRNA-transfected GM00637 cells as the control, the relative changes in the copy number of the miR146a-overexpressing GM00637 cells were measured. The chromosomal regions showing amplification or deletion are marked with a red line or a blue line, respectively. Schematic structures of chromosomes are displayed with their corresponding chromosome numbers. Array comparative genomic hybridization detected alterations of the clonal DNA copy number, including amplification in Chr3, Chr5, Chr6, Chr10, Chr15, Chr16 and Chr18, and deletion in Chr3, Chr5, Chr7, Chr9, Chr10, Chr14, and Chr15, in miR146a-overexpressing GM00637 cells.

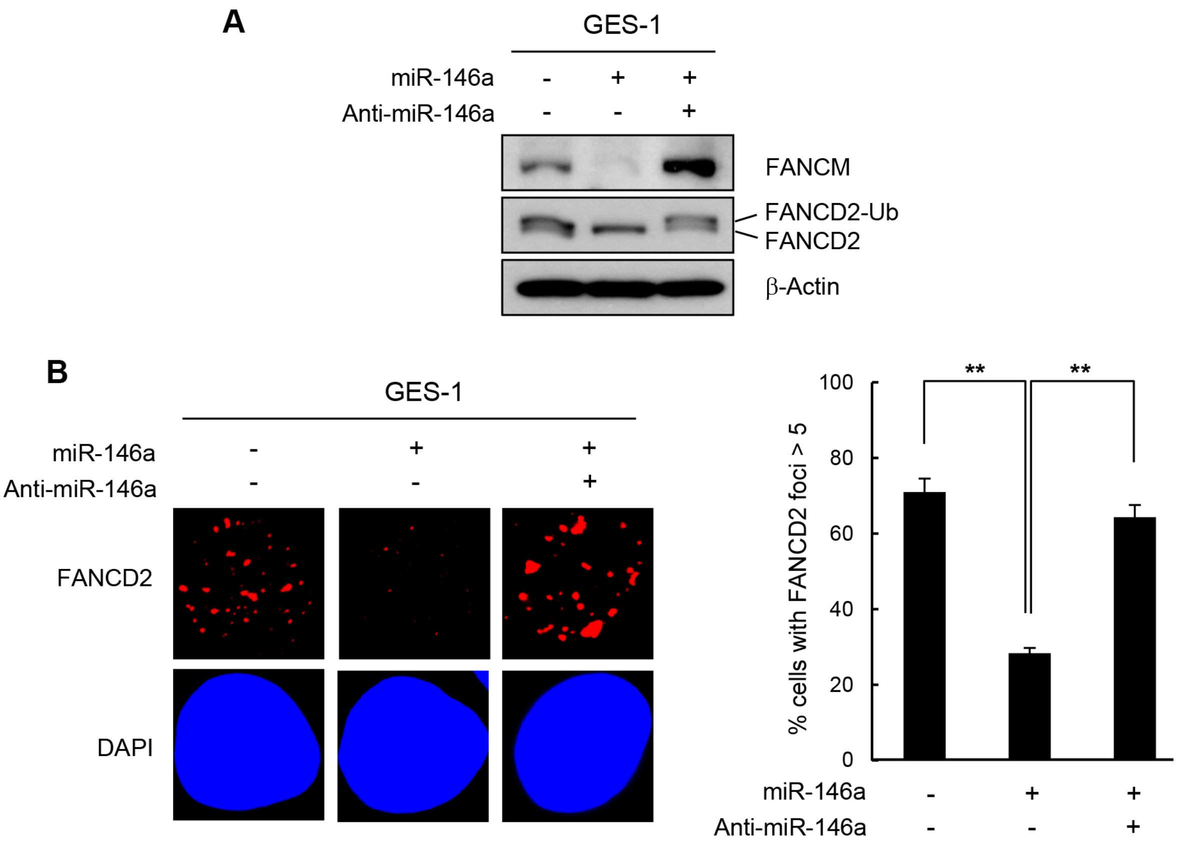

Supplementary Figure S6: Effect of miR146a on HU-induced FANCD2 activation. (**A**) GES-1 cells were transfected with miR146a in the absence or presence of anti-miR146a, After 48 h, the cells were treated with 5mM HU for 5h. Protein levels of FANCM and FANCD2 ubiquitination were measured by western blotting. β-Actin was used as loading control. (**B**) miR146a-expressing GES-1 cells were transfected with or without anti-miR146a and treated with 5mM HU for 5 h. Cells were analyzed for FANCD2 foci formation by immunostaining of FANCD2. DAPI was used for nuclear staining. Results are shown as the mean ± SD ($n = 3$); $^{**}P < 0.01$.

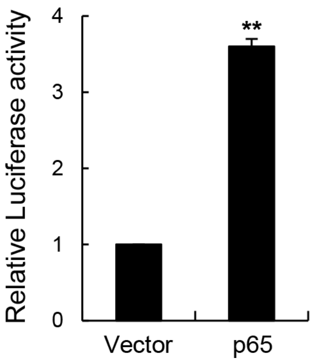

Supplementary Figure S7: NF-κB activation by overexpression of the p65/RelA subunit. HeLa cells were cotransfected with NF-κB promoter vector as reporter and pRL-TK vector containing *Renilla* luciferase as a transfection control in the absence or presence of p65 expression vector. 24 h after transfection, cell extracts were subjected to a luciferase reporter assay for detecting NF-κB activation. Results are shown as the mean ± SD (n = 3); **P < 0.01.

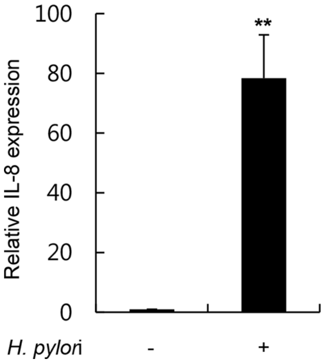

Supplementary Figure S8: H. pylori infection eliciting a host cell response. After GES-1 cells were infected with *H. pylori* for 24 h, the level of IL-8, a positive marker for host responses on *H. pylori* infection, was examined using RT-qPCR. Data are presented as the mean ± SD (n = 3); **P < 0.01.
